# Phase I study of adjuvant immunotherapy with autologous tumor-infiltrating lymphocytes in locally advanced cervical cancer

**DOI:** 10.1172/JCI157726

**Published:** 2022-08-01

**Authors:** He Huang, Cai-ping Nie, Xiu-feng Liu, Bin Song, Jian-hui Yue, Jing-xiao Xu, Jia He, Kui Li, Yan-ling Feng, Ting Wan, Min Zheng, Yan-Na Zhang, Wei-Jun Ye, Jun-Dong Li, Yan-Fang Li, Jun-yun Li, Xin-Ping Cao, Zhi-min Liu, Xiao-shi Zhang, Qing Liu, Xi Zhang, Ji-Hong Liu, Jiang Li

**Affiliations:** 1 State Key Laboratory of Oncology in South China, Collaborative Innovation Center for Cancer Medicine,; 2 Department of Gynecological Oncology,; 3 Department of Biotherapy, Sun Yat-sen University Cancer Center, Guangzhou, China.; 4 BGI-Shenzhen, Shenzhen, China.; 5 College of Life Sciences, University of Chinese Academy of Sciences, Beijing, China.; 6 Section of Cell Biology and Physiology, Department of Biology, University of Copenhagen, Copenhagen, Denmark.; 7 Department of Radiation Oncology, and; 8 Department of Cancer Prevention Research, Sun Yat-sen University Cancer Center, Guangzhou, China.

**Keywords:** Clinical Trials, Oncology, Adaptive immunity, Cancer

## Abstract

**BACKGROUND:**

Adoptive cell therapy (ACT) with tumor-infiltrating lymphocytes (TILs) has achieved remarkable clinical efficacy in metastatic cancers such as melanoma and cervical cancer (CC). Here, we explored the safety, feasibility, and preliminary tumor response and performed translational investigations of adjuvant immunotherapy using infusion of autogenous TILs (auto-TILs) following concurrent chemoradiotherapy (CCRT) in patients with CC who had locally advanced disease.

**METHODS:**

Twenty-seven patients with CC with stage III–IV disease were recruited in this single-center, phase I study. TILs were isolated from lesions in the uterine cervix and generated under good manufacturing practice (GMP) conditions and then infused after CCRT plus i.m. IL-2 injections.

**RESULTS:**

TILs from 20 of the 27 patients were successfully expanded, with a feasibility of 74.1%. Twelve patients received TILs following CCRT. Adverse events (AEs) were primarily attributable to CCRT. Only 1 (8.3%) patient experienced severe toxicity with a grade 3 hypersensitivity reaction after TIL infusion. No autoimmune AEs, such as pneumonitis, hepatitis, or myocarditis, occurred, and there were no treatment-related mortalities. Nine of 12 patients (75.0%) attained a complete response, with a disease control duration of 9–22 months. Translational investigation showed that the transcriptomic characteristics of the infused TIL products and some immune biomarkers in the tumor microenvironment and serum of patients with CC at baseline were correlated with the clinical response.

**CONCLUSION:**

TIL-based ACT following CCRT was safe in an academic center setting, with potentially effective responses in patients with locally advanced CC. “Hot” inflammatory immune environments were beneficial to the clinical efficacy of TIL-based ACT as adjuvant therapy.

**TRIAL REGISTRATION:**

ClinicalTrials.gov NCT04443296.

**FUNDING:**

National Key R&D Program; Sci-Tech Key Program of the Guangzhou City Science Foundation; the Guangdong Province Sci-Tech International Key Program; the National Natural Science Foundation of China.

## Introduction

Cervical cancer (CC) is the fourth most common cancer and represents one of the leading causes of cancer-related mortality in women worldwide, with approximately 570,000 new cases and 311,000 deaths annually (1). Concurrent chemoradiotherapy (CCRT) is the standard treatment for patients with locally advanced CC (2). However, the improvement in long-term outcomes seems to be more pronounced for patients with stage IB–IIB cancers than for those with stage III or IVA cancers (3). The prognosis for patients with advanced-stage disease remains poor, with 5-year survival rates for stage III and IVA of 39.3% and 24%, respectively (4), which highlights the need for novel therapeutic methods combined with CCRT as the primary treatment.

Adoptive cell therapy (ACT) using autologous tumor-infiltrating lymphocytes (auto-TILs) has been under development for melanoma treatment since the 1980s and can induce complete tumor responses in some patients (5–8). Recently, TIL-based ACT has been used to treat patients with HPV^+^ oropharyngeal, anal, or CC and has shown some clinical efficacy (9–11), which is worth further investigation. Recently, accumulating evidence has identified that TIL-based ACT treatment is beneficial for some metastatic cancers, including in some patients with checkpoint inhibition immunotherapy resistance (5, 12–14). However, some researchers have pointed out that TIL-based ACT might be used prior to other immunotherapies in eligible patients (15, 16). We have established the primary treatment pattern of TIL-based ACT combined with CCRT in patients with EBV^+^ nasopharyngeal carcinoma at advanced stages of disease and observed an objective clinical response and EBV-specific reactivity of T cells in some patients (17).

In this clinical trial, we first established an ex vivo “young” TIL expansion method under standard good manufacturing practice (GMP) conditions from transvaginally biopsied small tumor fragments. We sought to investigate the safety of this TIL-based ACT following CCRT in patients with CC with locally advanced disease; we also preliminarily evaluated feasibility and clinical activity. We evaluated correlates between immune parameters and clinical response to screen for potential biomarkers for the clinical benefit of TIL-based ACT as adjuvant therapy.

## Results

### Patients and feasibility

**Figure 1:**
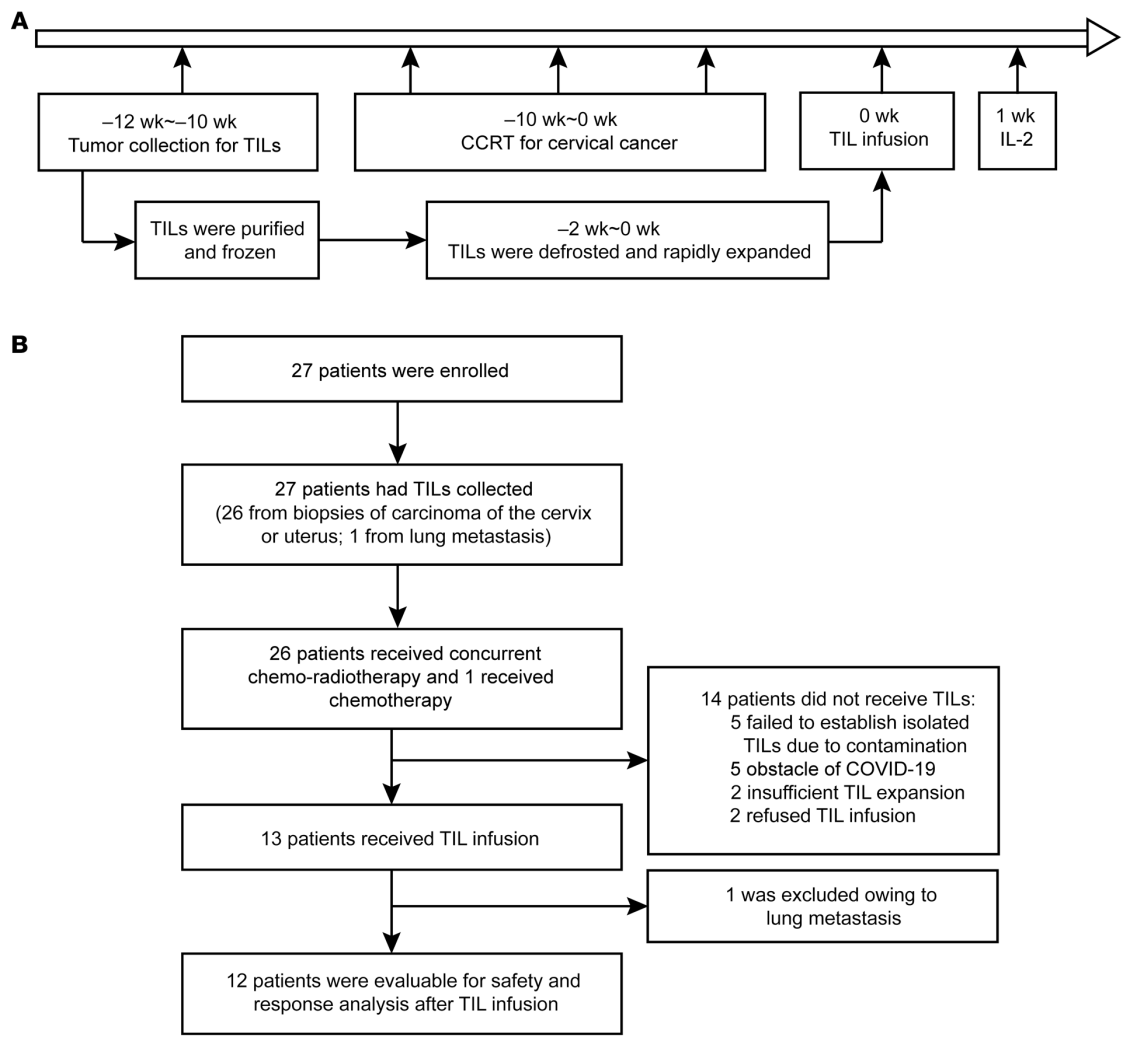
Schematic representing the study design and patient disposition. (**A**) Clinical trial schema. The week count is relative to TIL infusion. (**B**) Patient flow chart. Of the 27 patients enrolled, 13 patients received a TIL infusion after CCRT (*n* = 12 patients) or chemotherapy (*n* = 1 patient) and were evaluated for safety and tumor response. CCRT, concurrent chemoradiotherapy, radical radiotherapy for CC with concurrent cisplatin 30~40 mg/m^2^ weekly during EBRT.

A total of 27 patients with CC were enrolled between December 1, 2019, and December 17, 2020. The average age was 56 years (range, 42–70 years). Of the 27 patients, 24 were diagnosed with squamous cell carcinoma (SCC), and 3 were diagnosed with adenocarcinoma (AC). The International Federation of Gynecology and Obstetrics (FIGO) stages were III and IV (*n* = 25 in stage III and *n* = 2 in stage IV). Detailed patient information is shown in Supplemental Table 1; supplemental material available online with this article; https://doi.org/10.1172/JCI157726DS1 Biopsies of carcinoma in the cervix uteri (*n* = 26) and of metastatic cancer in the lung (*n* = 1) were collected. Purified lymphocytes were successfully obtained in 20 samples from 27 recruited patients, and expanded TILs were established ex vivo under GMP conditions, with a feasibility of 74.1% (20 of 27). The remaining 7 samples failed to establish ex vivo–expanded TILs because of contamination (5 of 27, 18.5%) and insufficient lymphocyte numbers for expansion (2 of 27, 7.4%). Among the 20 patients with successfully expanded TILs, 13 of them received an auto-TIL infusion plus i.m. IL-2 injection following CCRT. In total, 14 patients received CCRT treatment only (radical radiotherapy for CC and weekly cisplatin with external radiotherapy), including 2 patients who refused infusion, 5 patients who were hindered by the influence of COVID-19, and 7 patients who failed to establish expanded TILs, as shown in Figure 1.

Of the 13 patients who received an auto-TIL infusion plus an i.m. IL-2 injection, 11 tumors were classified as SCC and 2 were classified as AC (Tables 1 and 2). Among them, 12 patients received CCRT treatment and were included in the safety and efficacy analysis. Patient 1 was excluded because lung metastasis was found and diagnosed as stage IVB; thus, the patient did not undergo CCRT but received systemic chemotherapy first with paclitaxel and cisplatin instead, followed by auto-TIL infusion plus an i.m. IL-2 injection.

### Safety and adverse events

Adverse events (AEs) were mostly attributable to CCRT. The most common severe AEs were hematological and gastrointestinal toxicities during chemoradiotherapy in patients who received CCRT followed by TIL infusion. No treatment-related mortalities occurred. The toxicity profile was consistent with that of CCRT only (Table 3). Grade 1 or 2 toxicities were common and included nausea, vomiting, diarrhea and constipation. Fatigue was observed in 33.3% patients. Grade 3 or 4 toxicities were hematological during chemoradiotherapy. Anemia was the most common AE. No unexpected toxicity was observed, and all adverse reactions were manageable following standard guidelines.

Three AEs, including 1 (1 of 12, 8.3%) severe toxicity, were related to the TIL infusion. Patient 19 experienced a grade 3 hypersensitivity reaction 30 minutes after auto-TIL infusion, with a decrease in blood pressure, dizziness, and mild dyspnea. The symptoms resolved after intravenous administration of epinephrine and dexamethasone. According to prespecified criteria for the safety endpoint, this event was defined as severe toxicity. This patient achieved a complete regression 4 months after treatment. The other grade 1 or 2 AEs included 1 patient who had an allergic reaction with itchy skin and a mild rash and another patient experienced fatigue. No autoimmune AEs, such as pneumonitis, colitis, hepatitis, nephritis, or myocarditis, occurred, and there were no treatment-related mortalities. Seven patients (58.3%) experienced a low fever after the IL-2 injection; the symptoms resolved after the IL-2 injection, without any antipyretic treatment. The AEs for all patients with or without an auto-TIL infusion plus an i.m. IL-2 injection following CCRT are summarized in Table 3.

### Clinical activity

**Figure 2:**
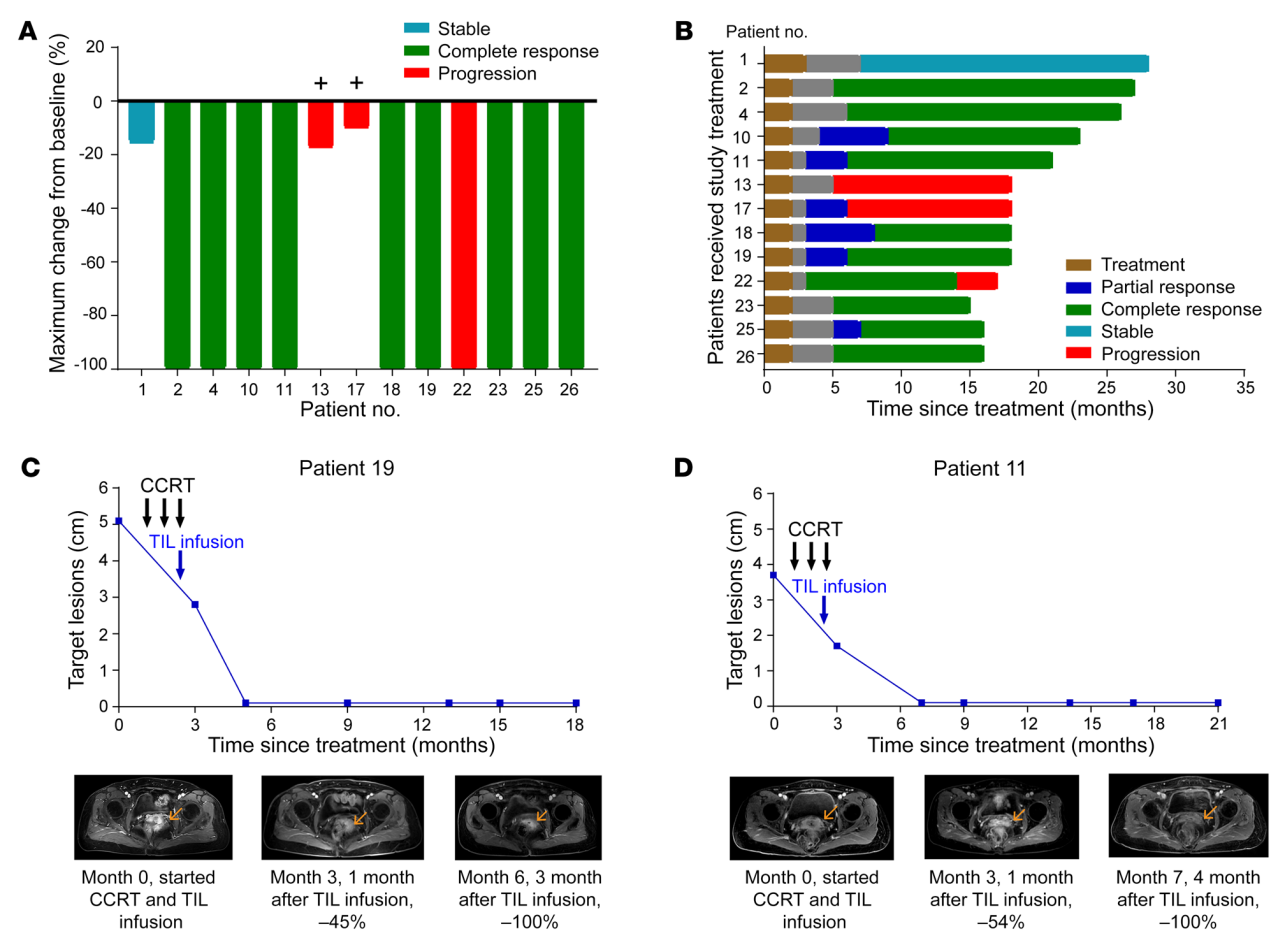
Clinical evaluation of patients for CCRT and auto-TIL treatment. (**A**) Waterfall plot of the maximum change in the sum of the target lesion (primary tumor lesion of the uterine cervix) compared with baseline measurements in 13 patients. “+” indicates distant metastasis. Patients 13 and 17 presented with distant lung and bone metastases. Patient 22 had pelvic recurrence after a 9-month CR. (**B**) Swimmer plots of the change in the sum of the target lesions from the treatment in 13 patients. Each bar represents 1 patient in this study. (**C** and **D**) MRI scans obtained at baseline and after CCRT and TIL infusion for CC patients 11 and 19.

Until the last follow-up on March 1, 2022, nine of the 12 patients who received a TIL infusion (75.0%) had complete regression of 1 or more tumors, with a disease control duration of 9–22 months (Table 1). Five patients (patients 2, 4, 22, 23, and 26) achieved a complete response (CR) 3 months after CCRT and TIL infusion; however, patient 22 experienced progressive disease (PD) after 14 months of a CR (Figure 2, A and B). The other 5 patients (patients 10, 11, 18, 19, and 25) experienced a partial response (PR) after 3 months of treatment and then attained a CR in the following 2–5 months, as shown in Figure 2, C and D, for patients 19 and 11, respectively. No deaths occurred among these 12 patients, and the mean progression-free survival (PFS) and overall survival (OS) times were 23 and 25 months, respectively.

Among the 14 patients who received CCRT, only 1 patient refused treatment and was lost to follow-up. Two patients died as a result of their disease 11 and 16 months after CCRT treatment. The death rate was 15.4% (2 of 13). Nine patients (9 of 13, 69.2%) achieved a CR, and 2 patients (2 of 13, 15.4%) had a PR (Table 2).

### Correlates between clinical response and immune parameters

#### Characteristics of infused TIL products.

**Figure 3:**
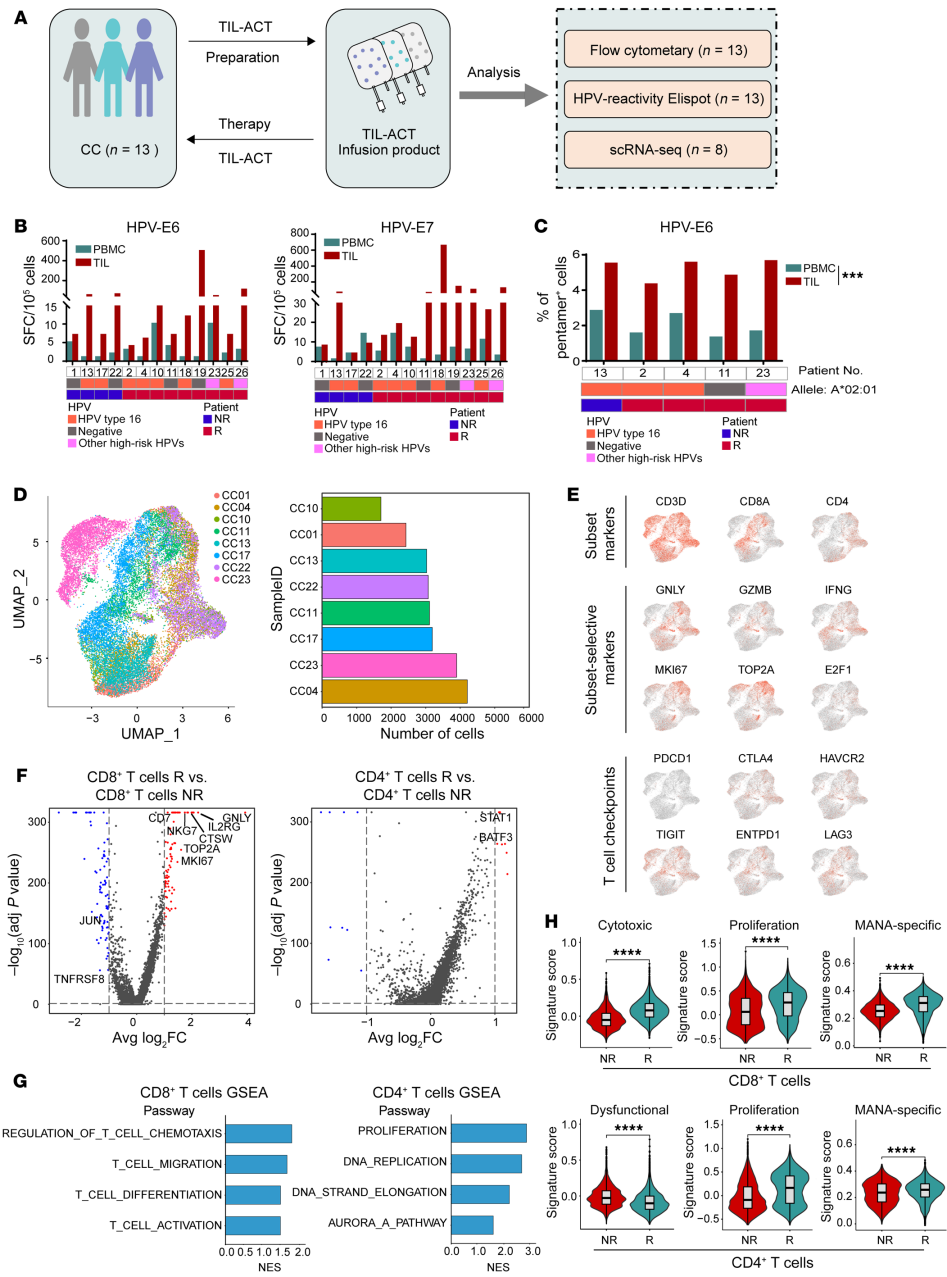
Correlations of characteristics of infused TIL products and clinical response. (**A**) Schematic illustration of biomarkers and functional identification of TIL products in this study. (**B**) Frequency of T cell reactivity against HPV E6 (left) and E7 (right) antigens in peripheral blood and TILs (*n* = 13). (**C**) Frequency of HPV E6 antigen–specific T cells in peripheral blood and in TILs from patients with HLA-A2^+^ CC (*n* = 5), by Wilcoxon test. (**D**) UMAP plot showing cells from 8 patients with CC. Bar graph shows the number of cells for each indicated patient (*n* = 8). (**E**) Expression and distribution of canonical T cell marker genes (CD3D, CD8A, and CD4) and genes related to cytotoxicity and proliferation among these cell subsets. (**F**) Volcano plots showing DEGs in CD8^+^ T cells (left) and CD4^+^ T cells (right) in responders versus nonresponders. Representative genes are labeled. adj, adjusted; avg, average; FC, fold change. (**G**) GSEA shows the pathway activities in CD8^+^ T cells (left) and CD4^+^ T cells (right) between responders and nonresponders. NES, normalized enrichment core. (**H**) Violin plots show the key signature scores of CD8^+^ T cells (top) and CD4^+^ T cells (bottom) (responders vs. nonresponders). ***P < 0.001 and ****P < 0.0001, by Mann-Whitney *U* test. R, responders; NR, nonresponders.

**Figure 4:**
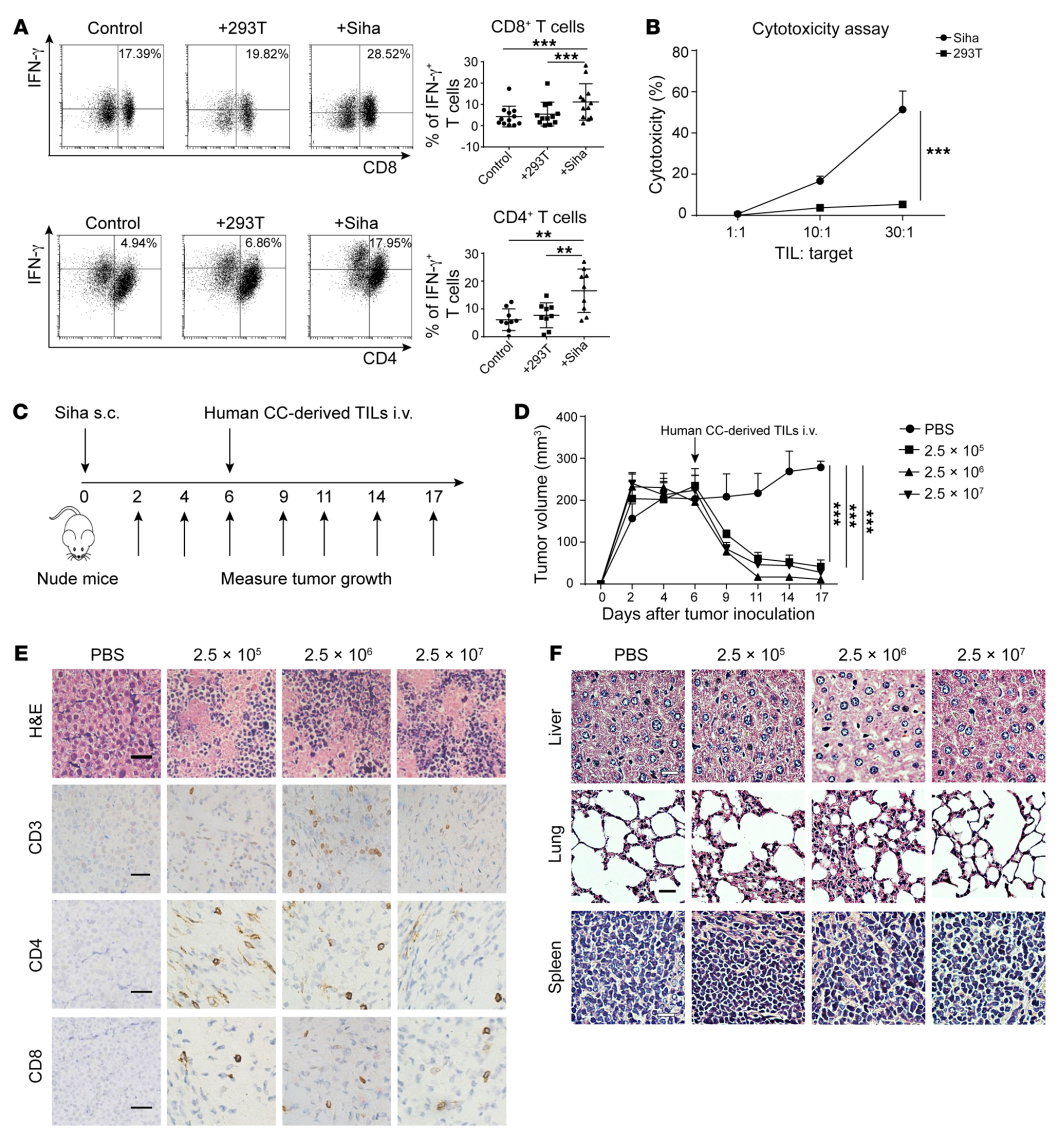
Specific cytotoxic effects and antitumor effects of TILs in vitro and in vivo. (**A**) Representative flow cytometric plots (left) and summary graphs (right) showing the frequencies of IFN-γ–producing T cells among CD4^+^ (*n* = 9) and CD8^+^ (*n* = 12) TILs cocultured with SiHa and 293T cells. (**B**) LDH cytotoxicity assay showing the specific killing effect of TILs (*n* = 3). (**C**) Experimental scheme for monitoring tumor growth and TIL therapy. (**D**) Time course of tumor growth in different groups adoptively transferred with different doses of human TILs isolated from patients with CC . *n* = 5. (**E**) Representative images of H&E staining of transplanted tumor tissue and representative IHC images of staining for anti–human CD3, CD4, and CD8 in the TME. (**F**) Representative images of H&E staining of liver, lung, and splenic tissue from nude mice in each experimental group. Scale bars: 100 μm. Data are shown as the mean ± SEM. **P < 0.01 and ***P < 0.001, by Mann-Whitney *U* test (**A**, **B**, and **D**).

We analyzed the biological characteristics of the infused TIL products by flow cytometry and IFN-γ ELISPOT array (*n* = 13) and single-cell scRNA-Seq (scRNA-Seq) (*n* = 8, Figure 3A). First, we observed that the reactivity of T cells against HPV E6 and E7 antigens was enriched in TIL products relative to circuiting T cells and that most TILs were composed of CD3^+^CD4^+^, CD3^+^CD8^+^, and CD3^+^CD56^+^ cells (Supplemental Figure 1, A–D). No associations were found between the frequency of E6- or E7-specific T cells and the composition of the TIL subset and patient clinical efficacy in this study (Figure 3, B and C). We further found that the majority of infused TILs were PD-1^+^Tim3^–^CXCR5^+^ central memory cells and that expression levels of CD137 were increased in the rapidly expanded infused TIL products (Supplemental Figure 1, C and D). Furthermore, scRNA-Seq analysis showed that cells from 8 TIL productions were interspersed across multiple clusters and defined as CD8^+^ and CD4^+^ cell clusters on the basis of filtered and normalized transcript counts. The cell clusters from the scRNA-Seq array were verified by FACS gating strategy analysis (Figure 3D and Supplemental Figure 1, E and F). Genes related to proliferation and cytotoxicity as well as T cell immune checkpoints were visualized in the CD3, CD8, and CD4 cell subsets (Figure 3E). Differentially expressed gene (DEG) analysis showed that genes related to cell differentiation and activation, including *CTSW*, *NKG7*, *GNLY*, *MKI67*, and *STAT1*, were expressed at high levels in responders compared with nonresponders (*n* = 4 and 4, respectively, Figure 3F). The activation and proliferation signaling pathways as well as the levels of cytotoxic, proliferative, and mutation-associated, neoantigen-specific (MANA-specific) T cell signatures were upregulated in responders’ TIL infusion products, but the dysfunctional cell signature was downregulated (Figure 3, G and H). We further determined the antitumor reactivity of the infused TIL products by detecting INF-γ release and cytotoxicity against SiHa (HPV^+^, partly MHC-matched) cells in vitro (Figure 4, A and B), as well as SiHa tumor growth inhibition in nude mice (Figure 4, C and D). Importantly, we found no observable toxicity in SiHa tumor–bearing nude mice infused with human TILs isolated from patients with CC (partly MHC-matched) and observed infiltration of the infused TILs into tumor tissues (Figure 4, E and F).

#### Peripheral and tumor immune parameters.

**Figure 5:**
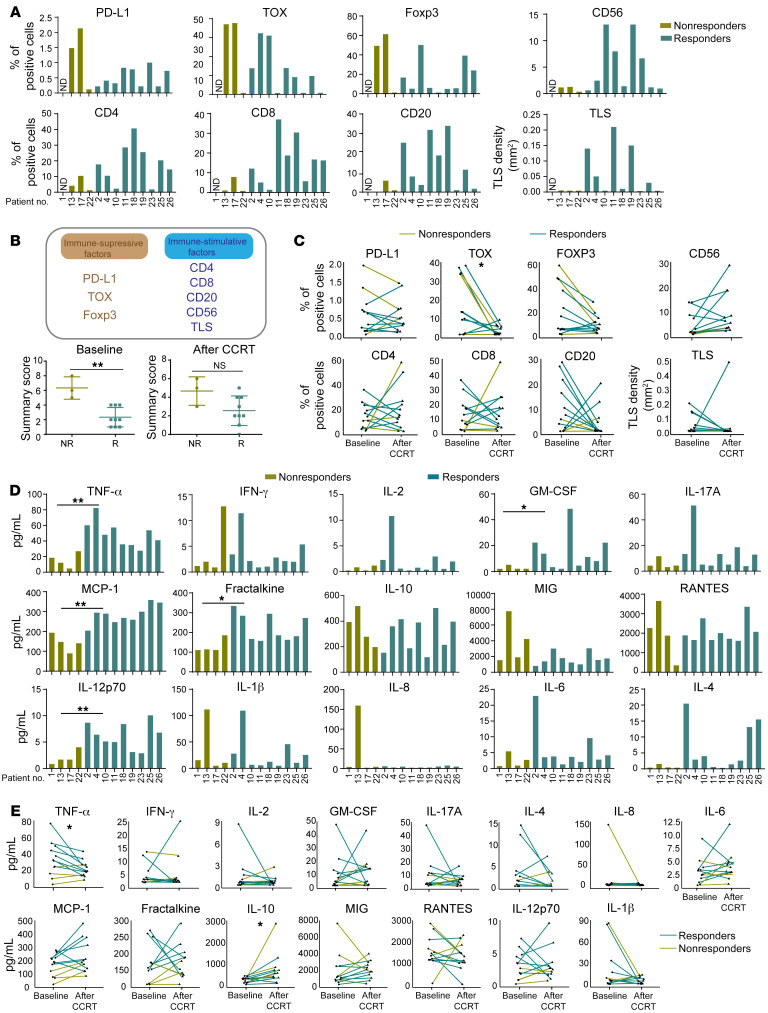
Linkage of baseline biomarkers and dynamic changes in biomarkers after CCRT to clinical response. (**A**) Percentage of positive cells with indicative biomarkers, including PD-L1, TOX, Foxp3, CD4, CD8, CD56, CD20, and TLSs in 12 tumor specimens from patients with CC at baseline (*n* = 9 responders and *n* = 3 nonresponders). (**B**) Immune factors (top) in the TME were divided into immune-suppressive factors (PD-L1, TOX, Foxp3) and immune-stimulative factors (CD4, CD8, CD20, CD56, TLS) according to the function of the gene or the indicated cell population (bottom). The combined immune score of PD-L1, TOX, Foxp3, CD4, CD8, CD20, CD56, and TLS at baseline (left) and after CCRT (right) in responders (*n* = 9) versus nonresponders (*n* = 3). The calculation of the combined immune score is described in Methods. (**C**) Changes in indicative biomarkers in CC specimens before and after CCRT (*n* = 12). (**D**) Histograms showing the serum levels of cytokines and chemokines, including TNF-α, fractalkine, IL-12p70, MCP-1, IFN-γ, IL-2, IL-1b, IL-17a, IL-4, IL-6, GM-CSF, RANTES, IP-10, IL-8, and MIG, at baseline in responders (*n* = 9) and nonresponders (*n* = 4). (**E**) Changes in indicative serum cytokines and chemokines in patients with CC at baseline versus after CCRT (*n* = 13). *P < 0.05 and **P < 0.01, by Mann-Whitney *U* test for nonparametric data. A paired Student’s *t* test was used to determine significance for all comparisons at baseline and after CCRT.

**Figure 6:**
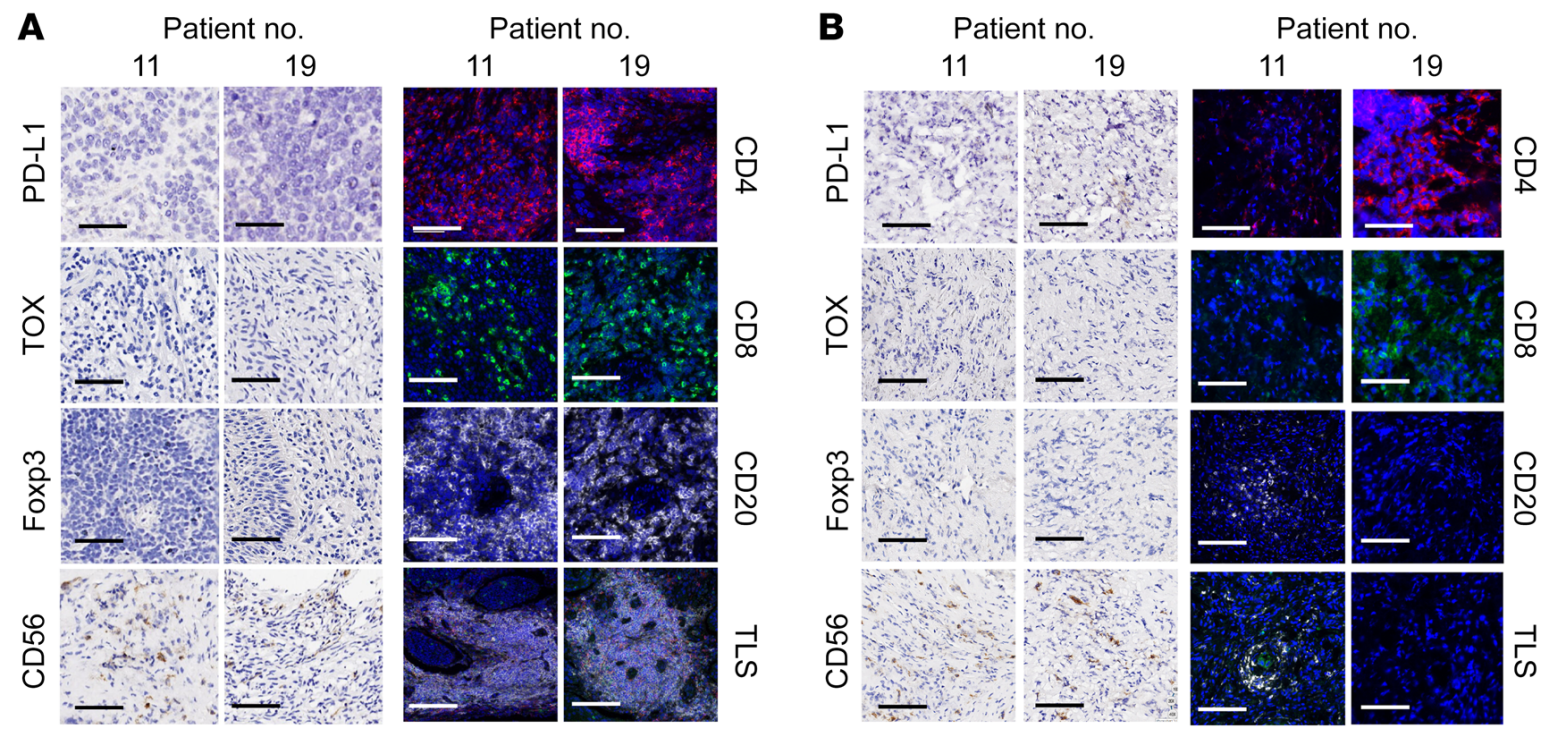
Immune evaluation for tumor microenvironments at baseline and after CCRT from 2 CC patients with CR. (**A** and **B**) Representative IHC and IF images of samples from patients 11 and 19 showing PD-L1, TOX, Foxp3, CD56, CD4 (red), CD8 (green), and CD20 (white) expression, and multiplex IF staining showing TLSs composed of CD20^+^, CD4^+^, and CD8^+^ cells at baseline (**A**) and after CCRT treatment (**B**). Scale bars: 50 μm and 100 μm for IHC and IF images, respectively. DAPI (blue) was used for nuclear staining. Original magnification, ×10.

In the exploration analysis, we further investigated feasible predictors for the clinical benefit of auto-TIL treatment based on the tumor and peripheral immune parameters of patients at baseline and after CCRT or TIL-based ACT treatment. We found that a combined immune score calculated on the basis of the levels of immune-inhibitory factors (programmed death ligand 1 [PD-L1], Thymocyte selection-associated HMG box protein [TOX], and Foxp3) and immune-stimulatory factors (CD4, CD8, CD20, CD56, and tertitary lymphoid structures [TLS]), as shown in Methods, revealed higher levels of these factors in nonresponders at baseline (P < 0.05) and that CCRT reduced the expression of immune-suppressive factors such as TOX (P < 0.05) and induced more infiltrated lymphocytes in tumor tissues (Figure 5, A–C). We did not observe an association between the alteration of peripheral HPV E6 or E7 antigen–specific T cells or immune cell subsets, including CD3^+^ T cells, CD3^+^CD4^+^ T cells, CD3^+^CD8^+^ T cells, CD3^–^CD16^+^ NK cells, CD4^+^CD25^+^Foxp3^+^ Tregs, PD1^+^CXCR5^–^Tim-3^–^ T stem (Tsm) cells, and PD1^+^CXCR5^+^Tim-3^+^ exhausted T (Tex) cells, and clinical efficacy in this trial (Supplemental Figure 2, A and B). However, the peripheral lymphocyte count was significantly decreased after CCRT compared with baseline (*P* < 0.05, Supplemental Figure 2C). Moreover, responders (*n* = 9) had higher baseline serum levels of inflammatory cytokines and chemokines, including TNF-α, IL-12, MCP-1, and fractalkine (CX3CL1) than did nonresponders (*n* = 3, *P* < 0.05, Figure 5D). Interestingly, CCRT increased the serum cytokine levels of IP-10 but decreased TNF-α levels (*P* < 0.05, Figure 5E). Overall, we observed “hot” microenvironments with lower levels of inhibitory factors (PD-L1, TOX, and Foxp3) and higher levels of infiltrated lymphocytes, including T cells, NK cells, B cells, and mature TLSs, in responders such as patients 19 and 11, who had a CR (Figure 6, A and B). Accordingly, “cold” microenvironments with higher levels of inhibitory factors and lower levels of infiltrated lymphocytes as well as low numbers of mature TLSs were observed in nonresponders such as PD patients 13 and 17 (Supplemental Figure 3).

## Discussion

In this trial, we proposed a primary treatment pattern of TIL-based ACT following CCRT in CC patients with advanced-stage disease (FIGO stage IIIA–IVA). For these patients, CCRT with cisplatin remains the standard treatment; however, the survival outcome is unsatisfactory (approximate 3-year OS rate of only 32%–45% for stage IVA) (3, 18–22). Thus, it is essential to search for novel therapeutic methods combined with CCRT in the primary treatment that would improve the prognosis. Immunotherapies, including immune checkpoint inhibitors or adoptive immune cells, have shown efficacy in the treatment of CC and may provide a longer tumor control period and better survival (23, 24). We successfully established a protocol for a GMP therapy–level TIL expansion approach in vitro using small biopsy samples obtained transvaginally from patients and aimed to determine the safety and feasibility of TIL-based ACT following CCRT as adjuvant treatment.

Most clinical trials for TIL-based ACT have been successfully launched in metastatic cancers such as HPV^+^ CC, lung cancer, and melanoma, and tumor regression has been observed in some cancers: the objective response rate (ORR) ranges from 28% to 50% and changes in cancers of different origins (5–7, 13, 25, 26). However, recently, some researchers have pointed out that the use of TIL-based ACT prior to other immunotherapeutic strategies in eligible patients may provide benefit in terms of the clinical response (15, 27); a randomized trial of auto-TIL–based ACT as adjuvant immunotherapy was reported in stage III melanoma without distant metastasis in 2002, and the researchers updated the follow-up period in 2007 and 2014. This study revealed that, after adjusting for tumor metastatic lymphoid node numbers, the patients who received auto-TIL–based ACT treatment had a longer relapse-free survival (RFS) and OS compared with the patients who received the IL-2 injection only (16, 28, 29). For locally advanced CC (FIGO stage I, stage II with tumor size larger than 4 cm, or stage IIB to IVA) treated with CCRT, the CR rate was reported to range from 62.5% to 81.3% (30, 31). In this study, TIL infusion following CCRT also induced a potent response, with a CR rate of 75% in patients with stage IIIA–IVA disease (disease control time, 9–22 months until the last follow-up) and median PFS and OS times of 23 and 25 months, respectively. The relatively longer disease control period may indicate the potential long-term benefit of auto-TIL infusion. Nevertheless, this clinical achievement should be confirmed in a large-sample, phase II study containing a control group with prognostic observations.

The toxicities that occurred during TIL therapy were mostly due to lymphodepleting preparative regimens and subsequent IL-2 injection after the TIL infusion. The toxicities related to the TIL infusion were less common and may include dyspnea, chills, and fever (32, 33). In our study, toxicities resulting in pancytopenia, gastrointestinal toxicity, and fatigue were predominantly caused by CCRT and were consistent with the toxicity profiles in patients treated with CCRT alone (34). One patient experienced a grade 3 allergic reaction related to TILs shortly after the infusion. The symptoms resolved after intravenous epinephrine and dexamethasone administration. Autoimmune toxicities, including vitiligo, hearing loss, or uveitis, were much less common. Uveitis usually responds well to local corticosteroid treatment (35). Overall, the observed toxicities were manageable for the most part. No specific safety signal of concern was identified for the cells themselves.

It has been mentioned that the roles of radiotherapy and chemotherapy in immune regulation are still controversial. It has been reported that radiotherapy mediates its antitumor effects at least in part by synergizing with the host immune system (36). Some studies have reported that radiotherapy can enhance TAA presentation by DCs to immune cells and enhance the recruitment of antitumor T lymphocytes, such as DCs and CD8^+^ T cells, in the tumor site by upregulating adhesion molecules (37). On the other hand, radiotherapy can directly inactivate immune cells and lead to the recruitment of myeloid-derived suppressor cells and Tregs in the tumor microenvironment (TME), promoting immune tolerance toward tumor cells (38, 39). Thus, it is a reasonable modality with CCRT followed by immunotherapy, such as ACT infusion. In addition, our previous phase I study of CCRT combined with TIL infusion in nasopharyngeal carcinoma showed that CCRT could induce lymphodepletion. In this study, CCRT was also set as a lymphodepletion treatment prior to TIL infusion in consideration of the rationality of the overall treatment scheme and the toxicity of the lymphodepletion regimen. A significant decrease in the lymphocyte count was observed after CCRT (Supplemental Figure 2C). Therefore, we did not implement lymphodepletion with cyclophosphamide and fludarabine, as described in other clinical trials for TIL-based ACT (40).

In addition to the safety and clinical response, we further explored the feasibility of establishing a TIL-based ACT strategy in patients with advanced CC. The process of isolating and manufacturing TILs is labor intensive and is only successful in a subset of patients (20%–40%); the process is usually restricted by the tumor excision location, size, and origin (41–44). However, we could isolate pure lymphocytes from most transvaginal biopsy samples, which were usually small in size (<0.5 cm in diameter), and only 2 of 27 samples failed to establish expanded TILs due to insufficient cell numbers. It is worth noting that the contamination caused by the open biopsy site (18.5%, 5 of 27) was a major difficulty in establishing successful TILs in this trial. These data suggest that the abundance of TILs in CC tissues allows for a therapeutic level of expanded TILs (>10^9^) to be obtained from small biopsy samples, but contamination should be prevented in tumor tissue procured by transvaginal biopsy. For infused TIL product assessment, infused TIL products contained higher levels of HPV E6 and E7 antigen–specific T cells, but we did not observe the correlation of the frequencies of HPV E6 and E7 antigen–specific T cells in TILs or peripheral blood and clinical response that was reported in another clinical trial of TIL treatment in HPV^+^ cancers (9–11). This result may be due to the small number of patients (several HPV^–^ patients were included, Supplemental Table 1) and the lack of some blood samples after TIL infusion because the patients contracted COVID-19. However, we demonstrated the function of HPV E6/E7 peptide–specific T cells against SiHa (HPV^+^) cells in vitro and in vivo (Supplemental Figure 4) and identified HPV E6/E7 as a potential target against CC. We observed distinct transcriptomic characteristics of the infused TIL products from responders and nonresponders by scRNA-Seq arrays, and the high level of gene clusters related to cytotoxicity, activation, and MANA-specific T cell signatures in infused TILs correlated with the clinical response. In addition, we further identified the function of infused TIL products by immune responses against SiHa (HPV^+^) cells in vitro and in vivo. These data suggest that TILs from patients with CC were composed of tumor or associated antigen–specific (neoantigen-specific) T cells and HPV antigen–specific T cells, both of which may contribute to tumor suppression in TIL-based ACT. Accordingly, immunotherapy based on checkpoint inhibition using anti–programmed cell death 1 (anti–PD-1) antibody therapy has archived outstanding clinical outcomes in patients with persistent, recurrent, or metastatic CC, who were also receiving chemotherapy (23, 45). These reported results indicate that CC tumors are highly immunogenic. Thus, TIL-based ACT combined with CCRT as an adjuvant to primary treatment may be a beneficial therapeutic strategy for patients with advanced CC.

We further explored the correlations between the clinical response and baseline immune-related biomarkers in this clinical trial. It has been reported that the levels of patients’ serum cytokines, tumor mutation burden, and immune checkpoints as well as the infiltrated immune cell composition may affect and predict the clinical achievement of TIL-based ACT (46–51). Here, we observed that low levels of immune-inhibitory factors, such as TOX and Foxp3, as well as high numbers of infiltrated lymphocytes in tumor tissues and high baseline levels of inflammatory cytokines may predict a clinical benefit for auto-TIL treatment. However, this finding needs to be confirmed in the near future in a large sample with more stringent statistical analysis. In summary, we found that TIL infusion after CCRT for locally advanced CC was feasible in an academic center setting and had effective responses with tolerable AEs, which suggests that further investigation of this type of therapy in the clinical setting in a wider population of patients with CC is worthwhile.

## Methods

### Study design.

This trial was a single-center, phase I study (ClinicalTrials.gov NCT04443296) that aimed to investigate the safety of cisplatin CCRT plus TIL infusion for the treatment of patients with FIGO stage IIIA–IVA CC.

Patients were treated with external beam radiotherapy (EBRT) at a dose of 45 Gy for the primary tumor and regional lymphatics at risk. The primary cervical tumor was then boosted using brachytherapy, with an additional 30–40 Gy, for a total dose of 85 Gy or higher. During EBRT, cisplatin was given weekly at 30–40 mg/m^2^ for a maximum of 6 doses. Ex vivo–expanded auto-TILs (>1 × 10^9^ cells in a single dose) were infused 3 days after the completion of CCRT and brachytherapy. After cell infusion, IL-2 was administered as an i.m. bolus at 400,000 IU/dose every 24 hours, for a total of 7 doses (Figure 1).

### Patients.

Patients from 18–70 years of age were eligible if they had SCC, AC, or adenosquamous carcinoma of the uterine cervix in FIGO stage IIIA–IVA. All patients planned to receive prior platinum-based chemoradiotherapy. An Eastern Cooperative Oncology Group performance status of 0 or 1 was required. The target lesion was defined as at least 1 detectable lesion by imaging.

### Assessments.

The primary objective of the study was to evaluate the safety of CCRT plus auto-TIL in treating patients with FIGO stage IIIA–IVA CC. AEs were recorded from the beginning of CCRT to 30 days following TIL infusion and graded according to the Common Terminology Criteria for Adverse Events (CTCAE), version 5.0. We aimed to evaluate 12 patients for toxicity in this study. Every 3 consecutive patients were treated as a cohort and evaluated for toxicity. If 1 or fewer severe toxicity events related to TIL infusion were observed in the first 3 patients, then 3 more patients were enrolled into the next cohort until 12 patients were included. If 2 or more patients within a cohort experienced severe toxicity events, then that study would be stopped. Severe toxicity was defined as grade 3 or higher nonautoimmune toxicity suspected to be related to TIL infusion (not related to CC or another preexisting condition in CCRT), or an autoimmune event that did not resolve with intervention (steroids) to grade 1 or lower within 21 days.

Secondary objectives included feasibility, primarily tumor response and its association with immunologic parameters, PFS, and OS. PFS and OS were defined as the time from treatment initiation until progression or death from any cause, respectively, or the date of data cutoff. Feasibility was defined as the rate of successful TIL generation from tumor biopsy specimens. Tumor response was evaluated according to the Response Evaluation Criteria in Solid Tumors (RECIST) version 1.1 guidelines. An objective response was defined as CR and a PR. Physical and imaging examinations (MRI/PET-CT/CT) were applied to determine the outcome at 1 month and every 3 months after the treatments.

### Generation of TILs.

Fresh tumor biopsy specimens were obtained from a transvaginal biopsy of the lesion and processed for the ex vivo expansion of “young” TILs. In brief, fresh tumor samples were collected in RPMI 1640 medium (Gibco, Thermo Fisher Scientific) with antibiotics, minced, enzymatically dissociated into single-cell suspensions with collagenase type IV (0.1 mg/mL, MilliporeSigma) and then plated into 24-well cell culture plates in X Vivo (Lonza) culture medium containing recombinant human IL-2 (1000 IU/mL) for 1 to 2 weeks to obtain purified T cells. Once a sufficient number of T cells (>10 × 10^6^) was generated, the cells were cryopreserved for further expansion. Clinical infusion products were generated by a rapid expansion protocol (REP) for “young” TILs: cryopreserved TILs were thawed and further expanded to numbers appropriate for treatment using a human anti-CD3 antibody (clone OKT-3, 30 ng/mL, R&D Systems), 3500 IU/mL human IL-2 (Sihuan Pharmaceutical), and irradiated feeder cells for 14 days under conditions in accordance with current GMP conditions in the Biotherapy Center at Sun Yat-sen University Cancer Center.

### scRNA-Seq for infused TIL products.

All steps from single-cell encapsulation to library preparation were performed at BGI-Shenzhen, following the manufacturer’s instructions. Single-cell capture, cDNA synthesis, and preamplification were performed using a DNBelab C4-V1 system (52). Libraries were sequenced on the MGISEQ2000 or DNBSEQ-T1&T5 platform. Raw scRNA-Seq data were processed using DNBelab C Series scRNA analysis software (https://github.com/MGI-tech-bioinformatics/DNBelab_C_Series_scRNA-analysis-software), including the gene expression data mapped to the human genome reference sequence (GRCh38). A number of steps were performed to filter out poor-quality data. First, cells with more than 200 expressed genes or greater than 15% of detected genes linked to mitochondrial genes were removed. Second, genes detected in fewer than 3 cells and cells with more than 7000 detected genes were filtered out. Third, the R package DoubletFinder (53) was applied to remove doublets, with an expected doublet rate of 0.04. For downstream analyses, the R package Seurat 4.0.0 (54) was applied to normalize the raw count matrix to identify highly variable genes, scale the genes, and integrate the samples. In addition, the first 20 principal components (PCs) and 2000 highly variable genes were used for unsupervised clustering analysis. The uniform manifold approximation and projection (UMAP) method performed by the RunUMAP function was used for dimensionality reduction and 2D visualization of the single-cell clusters. Clusters were labeled based on the canonical marker gene expression of the major cell type (CD8 and CD4). Differential expression analysis was performed using the FindMarkers function. Volcano plots were generated using the R package ggplot2 (55) for DEGs. Enrichment analysis to determine the signaling pathways in which the DEGs are involved was then carried out using gene set enrichment analysis (GSEA) with the R package clusterProfiler (56). Gene set scores of interest were calculated for each cell using the AddModuleScore function (57–59). The raw and processed single-cell sequencing data have been deposited in the NCBI’s Gene Expression Omnibus (GEO) database (GEO GSE190075).

### Flow cytometry.

The lymphocyte subsets and immune characteristics of infusion TIL products and the peripheral immune cells from patients with CC were detected by FACS staining and detection. Cells were washed twice using PBS, labeled with fixable viability dye (eBioscience/Thermo Fisher Scientific), and stained for biomarkers of interest using fluorochrome-conjugated antibodies (anti–human CD3, CD4, CD8, CD56, CD16, CD25, PD-1, TIM3, CXCR5, Foxp3, IFN-γ, and HLA-A*02:01-E618–26 pentamers) according to the manufacturer’s instructions. Detailed antibody information is shown in Supplemental Table 2. Intracellular Foxp3 and IFN-γ staining was performed with a fixation/permeabilization solution kit (BD Biosciences) following the instructions of the manufacturer. In brief, cells were stimulated with 10 ng/mL PMA (MilliporeSigma), 1 μg/mL ionomycin (Beyotime Biotechnology), and GolgiStop (BD Biosciences) in complete RPMI 1640 medium (Gibco, Thermo Fisher Scientific) for 4–6 hours, and then permeabilized and fixed for 1 hour, followed by Foxp3 or IFN-γ antibody staining. For intracellular staining of Foxp3, a fixation/permeabilization solution kit (BD Biosciences) was used following the instructions of the manufacturer. For intracellular cytokine IFN-γ staining, TILs were cultured for 4–6 hours with 10 ng/mL PMA (MilliporeSigma) and 1 μg/mL ionomycin (Beyotime Biotechnology) and GolgiStop (BD Biosciences) in RPMI 1640 medium. Then, the eBioscience Invitrogen Intracellular Fixation and Permeabilization Buffer Set (Thermo Fisher Scientific) according to the manufacturer’s instructions. The frequency of HPV E6–specific reactive T cells in infusion TIL products and peripheral blood was determined by bound HLA-A*02:01-E618–26 pentamers (peptide sequence: KLPQLCTEL; ProImmune), and at least 105 cells were captured by a FACS instrument for pentamer detection. Data were acquired with a Beckman Coulter flow cytometer and analyzed with FlowJo software (BD). Analyses were gated on live, singlet lymphocytes. Details on the antibodies and reagents used are provided in Supplemental Table 2.

### T cell functional assays.

The frequency of HPV E6– and E7–specific reactive T cells in the peripheral blood of patients at baseline, before and after TIL infusion, and in the infused TIL products was measured using a human IFN-γ–precoated ELISPOT PRO Kit (Da Ke Wei) according to the manufacturer’s instructions. T cells were incubated in this plate at 1 × 10^5^ cells per well and stimulated with 50 ng/mL of the E6 and E7 proteins (Miltenyi Biotec) or with autologous phytohemagglutinin-stimulated (PHA-stimulated) blast cells as a control for 20 hours at 37°C. ELISPOTs were developed using AEC plus (Dakewe) and counted automatically using ImmunoSpot 5.0.3 analysis software. Spot forming cells (SFC) indicate the number of IFN-γ–producing cells per 1 × 10^5^ cells after HPV E6/E7 stimulation.

### Generation of HPV E6- or E7–specific T cells.

We generated HPV-E6/E7 peptide–specific T cells in vitro using the following protocol. In brief, PBMCs were isolated from healthy donors and stimulated with 1 μg/mL of the E6 and E7 peptides (Miltenyi Biotec) in X-VIVO medium (Lonza) with 1500 IU/mL IL-2 (Sihuan Pharmaceutical) in an OKT3-precoated 24-well plate for 7 days, restimulated with 1 μg/mL of E6 and E7 peptides, and cultured for another 7 days. On day 14, HPV E6/E7 peptide–specific T cells were harvested and analyzed for flow cytometry, LDH cytotoxicity, and animal experiments.

### Lactate dehydrogenase assays.

The cytotoxicity of infused TIL products or HPV E6/E7–specific T cells was measured using a lactate dehydrogenase (LDH) assay. The infused TIL products from HLA-matched patients were cocultured with SiHa or 293T cells for 6 hours, and the cell supernatants were collected. LDH activity was measured using an LDH detection kit (MilliporeSigma) following the manufacturer’s instructions. The data were assessed by optical absorbance on a microplate reader at 490 nm. Cytotoxicity was calculated by the following formula: cytotoxicity percentage = (test sample – negative control)/(lysate control – negative control) × 100%.

### Xenograft mouse model.

The in vivo experiments were performed using 4-week-old female nude athymic mice (BALB/c-nu/nu, Vital River). In brief, after mycoplasma detection by PCR analysis, 5 × 10^6^ SiHa cells (mycoplasma negative) were resuspended in 100 μL PBS and injected subcutaneously into the axilla of the right upper limb. Approximately 1 week after transplantation, HLA-matched TILs from patients with CC or HPV E6/E7-specific T cells (2.5 × 10^5^, 2.5 × 10^6^, and 2.5 × 10^7^ cells) were injected intravenously into the tail vein for treatment. A xenograft plus PBS group was included as a control. Tumor growth was monitored every 3 days, and the tumor volume was calculated using the following formula: V = W2 × L/2, with W representing the shortest diameter and L the longest diameter. Then, the mice were sacrificed on day 17. The tumor node, lung, spleen, and liver were removed and weighed and fixed in 10% buffered formalin for histological examination. All mouse experiments were performed with groups of 5–6 mice. The mice were randomly grouped into the treatment or corresponding control groups, and the operators were blinded to the group assignments.

### Patient immune parameter analysis.

Tumor specimens (*n* = 12) and peripheral blood (*n* = 13) were collected from 13 patients who underwent TIL-based ACT treatment at baseline and after CCRT and/or auto-TIL treatment. The peripheral immune subsets were detected by flow cytometry, the serum cytokine profile was measured using the cytokine Milliplex assay, and the TME biomarkers were analyzed by immunohistochemistry (IHC) and immunofluorescence (IF).

### Serum cytokine profile analysis.

Serum cytokine and chemokine levels in serum were measured using a Cytokine Milliplex Assay Kit and a MAGPIX Multiplexing System (both from MilliporeSigma) following the manufacturer’s protocol.

### IHC and IF.

Formalin-fixed, paraffin-embedded tissue sections were continuously sectioned at a thickness of 4 μm, and an IHC kit (Zhongshan Jinqiao Biotechnology) was used according to the manufacturer’s instructions. In brief, tissue sections were deparaffinized and rehydrated by immersion in EDTA (pH 8.0) or 1× citrate (pH 6.0). A pressure cooker (95°C, 22 min) was applied for antigen retrieval. Goat serum was applied to block nonspecific binding sites at room temperature for 30 minutes. Primary antibodies, including anti–human PD-L1, anti-TOX, anti-Foxp3, and anti-CD56 antibodies, were incubated at 4°C overnight. The secondary antibody (Zhongshan Jinqiao Biotechnology) was incubated for 30 minutes at room temperature. DAB was used for visualization. Finally, the pathological sections were counterstained with hematoxylin and then dehydrated and sealed with neutral glue for optical microscopy.

For IF staining of TILs, an Opal Polaris 7 color manual IHC kit (Akoya Biosciences) was used following the manufacturer’s protocol with primary antibodies, including anti–human CD4, -CD8, and -CD20 antibodies. DAPI was used for nuclear staining and section mounting. Images were acquired using a PerkinElmer Vectra version 3.0 system, and Vectra software (Akoya Biosciences) and HALO software (Indica Labs) were used to analyze the images. Lymphocyte density was quantified as the percentage of cells expressing a given marker of at least 5 high-power fields (HPFs). Mature TLSs correspond to lymphoid follicles, including a dense cellular aggregate resembling germinal centers found in secondary lymphoid structures (SLOs). Calculation of the immune combined score was done as follows: 0 = low expression of PD-L1, TOX, or Foxp3 or high expression of CD4, CD8, CD20, CD56, or TLS; 1 = high expression of PD-L1, TOX, or Foxp3 or low expression of CD4, CD8, CD20, CD56, or TLS. The score was calculated by adding the expression of individual markers. The maximum score was 8.

### Statistics.

The data analysis was mainly descriptive. Summary statistics for AEs, including the proportions of each preferred AE type were tabulated and assembled into tables. AEs were categorized by grade. All correlative study results were treated as exploratory in nature, given the pilot status and sample size of the trial. Objective responses were plotted on applicable “waterfall” plots using the percentage of change. For correlative analysis, we explored the extent to which changes between pre- and post-treatment levels correlated with response by Student’s *t* test or Mann-Whitney *U* test. Furthermore, 2-tailed Student’s *t* tests, paired Student’s *t* tests, or Wilcoxon tests were performed for comparisons of 2 groups. A *P* value of less than 0.05 was considered significant. All statistical analyses were performed using R software (version 4.0.3), GraphPad Prism 5 (GraphPad Software), or SPSS 18.0 software.

### Study approval.

This clinical trial is registered at https://register.clinicaltrials.gov (ClinicalTrials.gov NCT04443296). All patients provided written informed consent independently and agreed to donate specimens for scientific study. This study was approved by the IRB of the Sun Yat-sen University Cancer Center (SYSUCC, B2019-124-01) and was conducted in compliance with the Declaration of Helsinki and Good Clinical Practice guidelines. All animal experiments were performed in accordance with protocols approved by the IACUC of Sun Yat-sen University, Guangzhou, China (L102012018060P).

### Data and materials availability.

The study protocols are provided in the Supplemental materials. The key processed and clinical data have been deposited in the Research Data Deposit public platform (www.researchdata.org.cn; accession code RDDA2022500226) to validate the authenticity of this study.

## Author contributions

JL, HH, and JHL conceived, designed and supervised the project. JL, HH, and QL contributed to the implementation and design of the clinical study, writing of the protocol, and statistical analysis and interpretation. XFL, BS, JHY, and XZ contributed to the scRNA-Seq and bioinformation analysis. CPN, JXX, JH, and KL participated in the generation and expansion of TIL-infused product as well as laboratory testing. YLF, TW, MZ, YNZ, WJY, JDL, YFL, JYL, XPC, ZML, and XSZ contributed to the recruitment and treatment of patients, data and trial management, and review of the report. HH, JL, CPN, XZ, and XFL were involved in the writing and revision of the manuscript. All authors have read and approved the manuscript. The order of the co–first authors was determined on the basis of their efforts and contributions to the manuscript.

## Supplementary Material

Supplemental data

ICMJE disclosure forms

## Figures and Tables

**Table 3:**
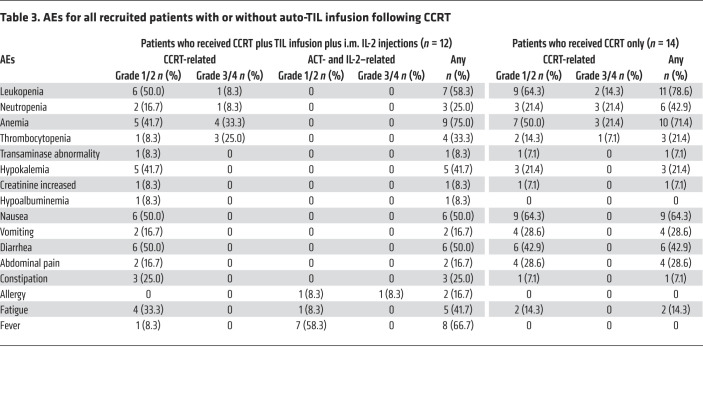
AEs for all recruited patients with or without auto-TIL infusion following CCRT

**Table 2:**
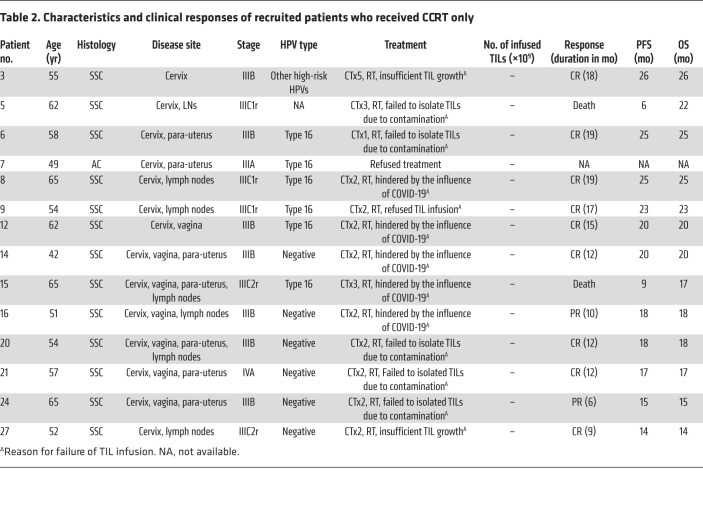
Characteristics and clinical responses of recruited patients who received CCRT only

**Table 1:**
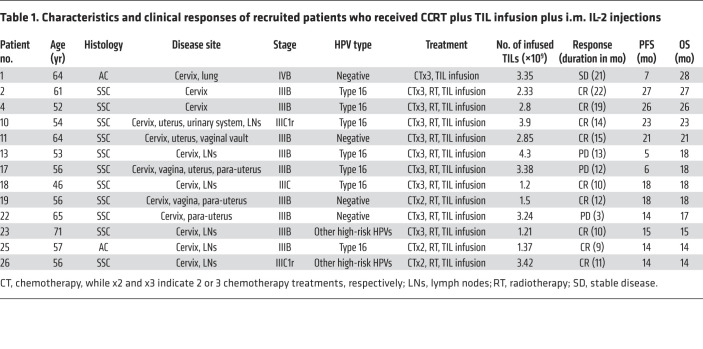
Characteristics and clinical responses of recruited patients who received CCRT plus TIL infusion plus i.m. IL-2 injections

## Version 1 06/21/2022

In-Press Preview

## Version 2 08/01/2022

Electronic publication
