# Supplementary material for: Phase I study of adjuvant immunotherapy with autologous tumor-infiltrating lymphocytes in locally advanced cervical cancer
Source: J Clin Invest. 2022 Aug 1;132(15):e157726. doi: 10.1172/JCI157726 (PMC9337833; doi:10.1172/JCI157726)
Supplement: Supplemental data [file jci-132-157726-s083.pdf]

Supplemental

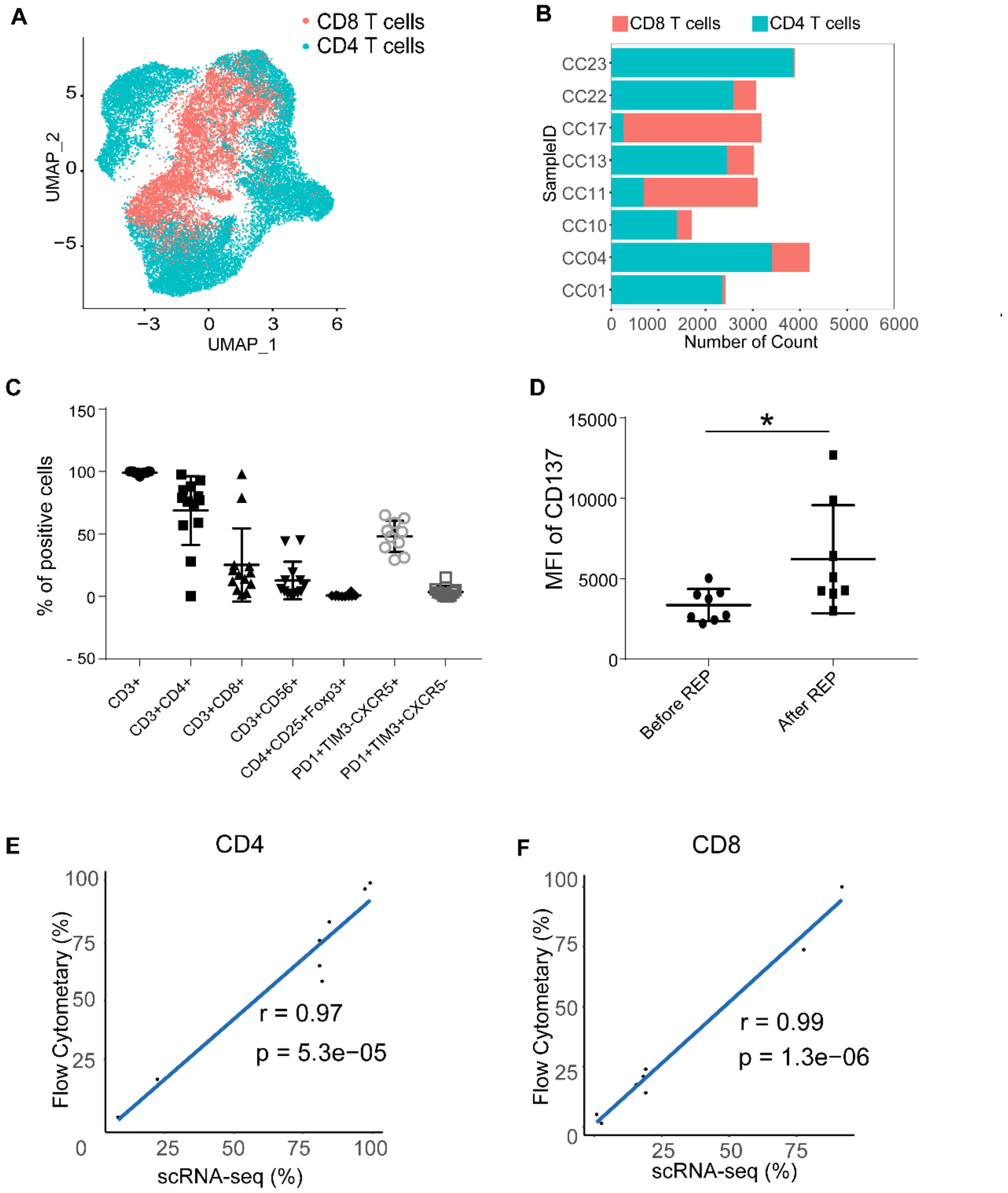

Supplemental Figure 1. Biological characteristics of infused TIL products were verified by scRNA-seq and flow cytometry. (A) UMAP plot showing cells from 8 TIL samples from CC patients colored by the major cell type. **(B)** Bar graph showing the number of cells and the distribution of CD4 and CD8 cells in infused TIL products from the corresponding patient origin. **(C)** Percentages of TIL subsets, including $CD3^{+}$

T cells, $CD3^+CD4^+$ T cells, $CD3^+CD8^+$ T cells, $CD3^+CD56^+$ NKT cells, $CD4^+CD25^+Foxp3^+$Tregs, PD-1^+CXCR5^+Tim3^-Tsm, and PD-1^+CXCR5^-Tim-3^+Tex, using flow cytometry analysis in ACT patients. All subsets were gating in $CD3^+$ cells (n = 10). **(D)** Summary graphs showing the mean fluorescence intensity (MFI) of CD137 in $CD8^+$ cells of TIL production before and after the rapid expansion protocol (REP) by flow cytometry (n = 8). *$p < 0.05$, Mann–Whitney test. **(E)** Dot plot showing the correlations for the fractions of $CD4^+$ T cells from 8 TIL-ACT infusion products using flow cytometry compared to the fractions measured using scRNA-seq. **(F)** Dot plot showing the correlations for the fractions of $CD8^+$T cells from 8 TIL-ACT infusion products using flow cytometry compared to the fractions measured using scRNA-seq.

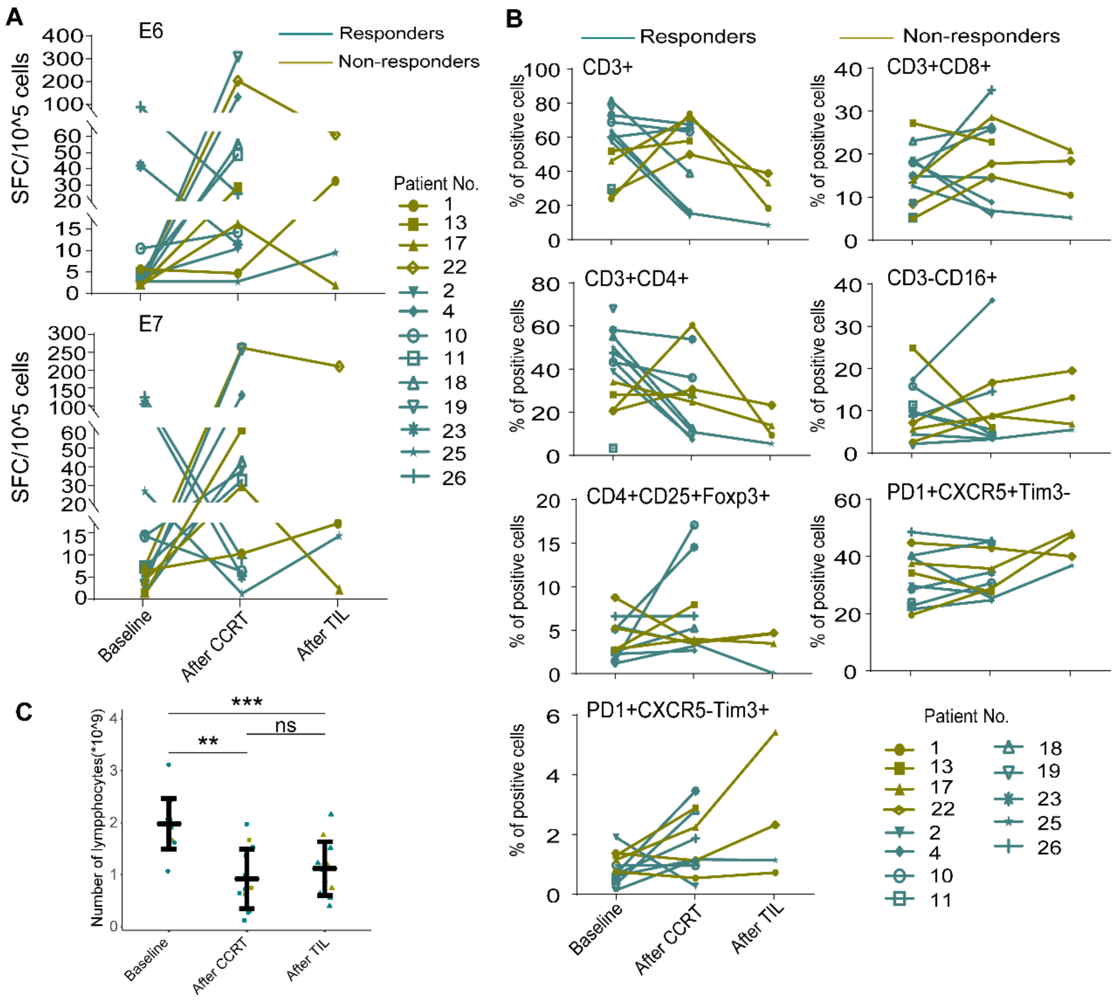

Supplemental Figure 2. Frequencies of circulating immune cell subsets and HPV E6/E7-reactive T cells in CC patients at baseline and after CCRT or TIL treatment. **(A)** PBMCs from patients at the indicated time points were assessed by IFN-γ ELISPOT assay for reactivity against HPV E6 (left) and E7 (right) antigens in individual patients who accepted CCRT and TIL treatment. Values shown represent the sum of HPV E6- or E7-specific spot-forming units (SFUs) after negative subtraction. The indicative lymphocyte subsets were determined by FACS staining and a multiplex gating strategy (n = 13). **(B)** The change in peripheral lymphocyte subsets, including $CD3^{+}$ T cells, $CD3^{+}CD4^{+}$ T cells, $CD3^{+}CD8^{+}$ T cells, $CD3^{-}CD16^{+}$ NK cells, $CD4^{+}CD25^{+}Foxp3^{+}$Tregs, PD-$1^{+}CXCR5^{+}Tim3^{-}$Tsm, and PD-$1^{+}CXCR5^{-}$Tim-3^{+}Tex, assessed at the indicated time

points in each patient who underwent CCRT and TIL treatment (n = 13). **(C)** The change in the number of lymphocytes in peripheral blood from patients at the indicated time points (n = 13).

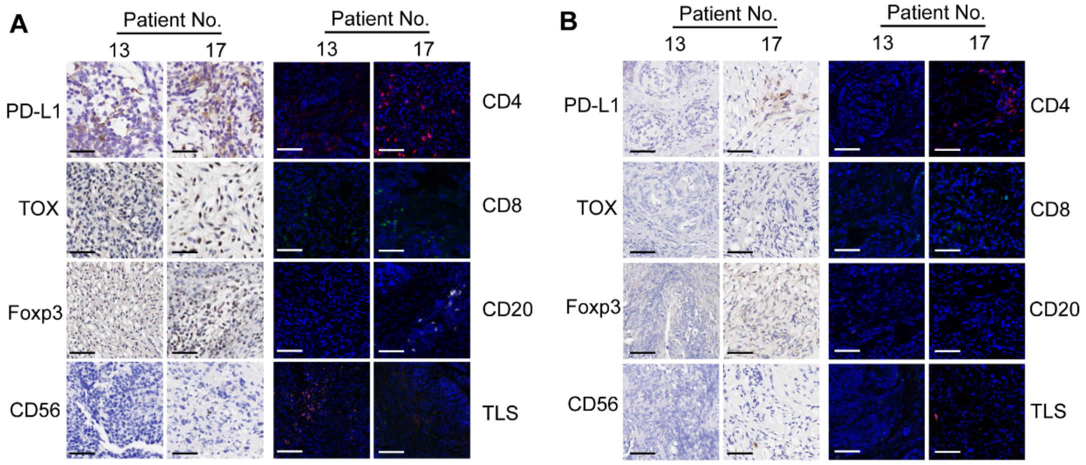

C

Patient No.	13		17	
	Baseline	After CCRT	Baseline	After CCRT
PD-L1[a]	1.48	0.86	2.13	1.61
TOX[b]	37.10	0.05	37.70	5.86
FOXP3[b]	49.28	11.25	61.26	19.64
CD56[b]	1.18	1.69	1.25	2.74
CD4[b]	4.06	0.37	10.38	14.03
CD8[b]	1.00	0.41	7.87	3.10
CD20[b]	0.00	0.04	5.83	0.05
TLS[c]	0.00	0.00	0.00	0.00

a. % of positive cells in tumor cells
b. % of positive cells in lympocytes
c. ratio of TLS area/tissue area

Supplemental Figure 3. Biomarkers in tumor tissues from CC patients with partial disease progression. (A-B) (left) Representative IHC and IF images of the indicated biomarkers of Patient Nos. 13 and 17 at baseline (A) and after CCRT (B). Scale bar denotes 50 μm. CD20 (white), CD4 (red), CD8 (green), and DAPI (blue). Original magnification, ×10. Scale bar denotes 100 μm. **(C)** Expression scores of indicative biomarkers in Patient Nos. 13 and 17 at baseline and after CCRT.

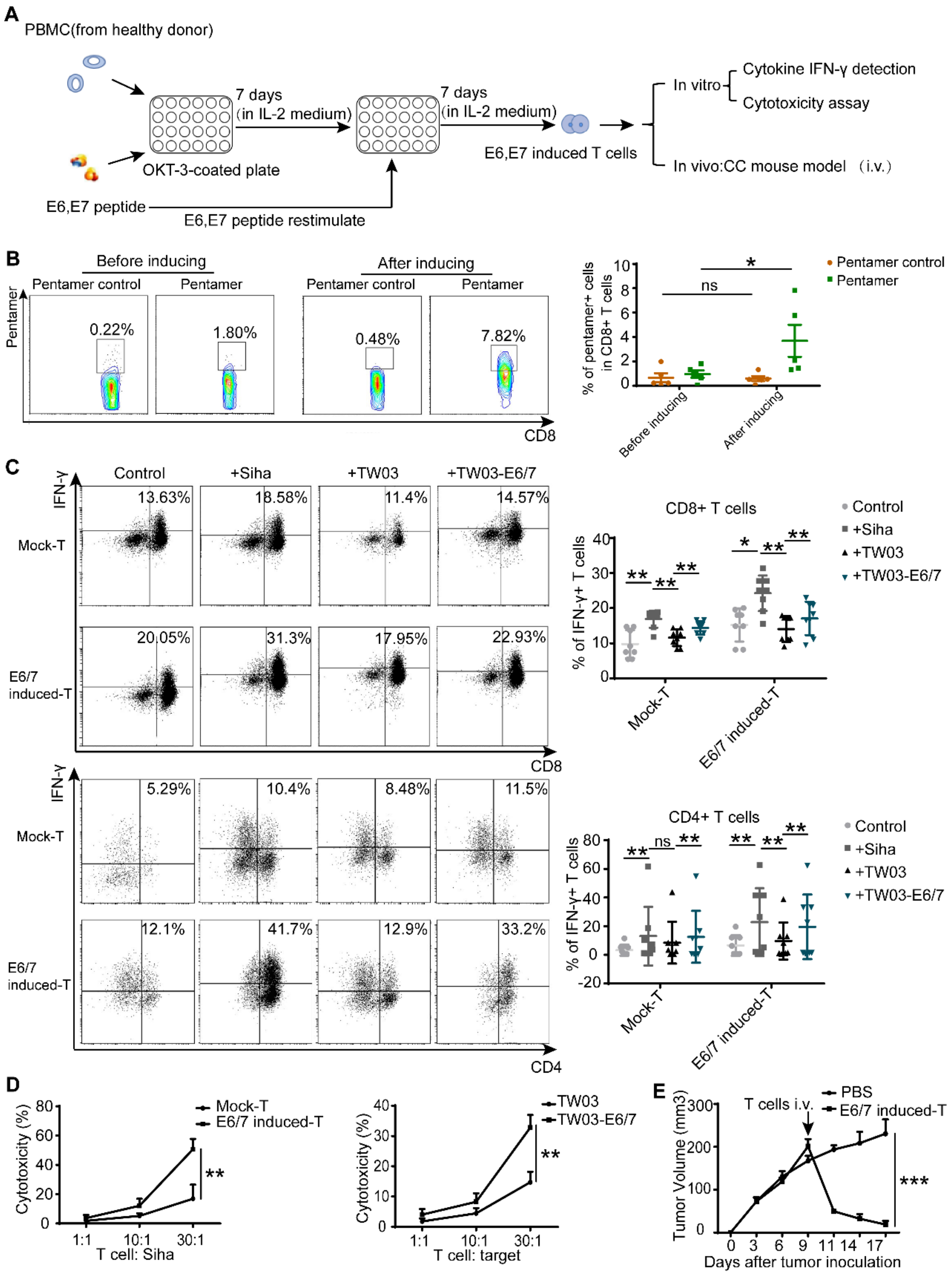

Supplemental Figure 4. Specific cytotoxic effects and antitumor effects of E6 and E7 antigen peptide-specific T cells in vitro and in vivo. (A) Scheme of E6- and E7-inducing T cells in vitro. **(B)** Representative flow cytometry plots (right) and summary graphs (left) showing the frequency of HPV E6 antigen-specific T cells

before vs. after induction (n = 5). **(C)** Representative flow cytometry plots (right) and summary graphs (left) showing the expression frequencies of IFN-γ among $CD4^+$ and $CD8^+$ E6/7-induced T cells and noninduced T cells cocultured with SiHa, TW03 and TW03 loaded with E6 and E7 peptide, respectively (n = 8). **(D)** LDH cytotoxicity assay showing the killing effect of E6/7-induced T cells. Target cells: SiHa (left), TW03 and TW03 loaded with E6 and E7 peptides (right) (n = 6). **(E)** Time course of tumor growth in different groups adoptively transferred with E6/7-induced T cells and PBS (n = 5). Data are the mean ± s.e.m. **P ≤ 0.01, Mann–Whitney test.

Supplemental Table 1. Patient Characteristics

Characteristic	No. (%) of patients
**Total cases**	27 (100%)
Age	56 (42-70)
**Sex**	
Female	27 (100%)
Male	0 (0%)
**Race or ethnic group**	
Asian	27 (100%)
Other races	0 (0%)
**Diagnosis type**	
SCC	24 (88.89%)
AC	3 (11.1%)
**FIGO stage**	
III	25 (92.59%)
IV	2 (7.41%)
**HPV type**	
HPV 16	14 (51.85%)
HPV 18	0 (0%)
Other high risk type*	6 (22.23)
Negative	5 (18.52)
NA	2 (7.4%)
**Treatment**	
CCRT	14 (51.85%)
CCRT+TIL	12 (44.44%)
CT+TIL	1 (3.71%)

Note: *Other type, HPV 31, 33, 35, 39, 45, 51, 52, 56, 58, 59 and 68; NA, not available.

Abbreviations: AC, adenocarcinoma; SCC, squamous cell carcinoma; CCRT, concurrent chemoradiotherapy; CT, chemotherapy.

Supplemental Table 2. Antibody Information

Antibody name	Application	Source	Host	Identifier	Clone	Dilution
FITC anti-human CD3	Flow cytometry	BioLegend	Mouse	300306	HTT3α	1:50
Bright Violet 610 anti-human CD4	Flow cytometry	BioLegend	Mouse	317438	OKT4	1:50
Bright Violet 650 anti-human CD8	Flow cytometry	BioLegend	Mouse	344730	SK1	1:50
eFluor™ 450 anti-human CD16	Flow cytometry	eBioscience	Mouse	48-0168-42	eBioCB16 (CB16)	1:50
PerCP-Cyanine7 anti-human CD56	Flow cytometry	eBioscience	Mouse	25-0567-42	CMSSB	1:50
PE anti-human CD25	Flow cytometry	BioLegend	Mouse	356104	M-A251	1:50
PE-Cyanine7 anti-human PD1	Flow cytometry	Invitrogen	Mouse	25-2799-42	eBioJ105	1:50
Bright Violet 510 anti-human TIM3	Flow cytometry	BioLegend	Mouse	345030	F38-2E2	1:50
Bright Violet 785 anti-human CXCR5	Flow cytometry	BioLegend	Mouse	356936	J252D4	1:50
APC anti-human Foxp3	Flow cytometry	Invitrogen	Rat	17-4776-42	PCH101	1:50
Bright Violet 510 anti-human IFNγ	Flow cytometry	BioLegend	Rabbit	506538	B27	1:50
BV510 anti-human CD137	Flow cytometry	BD Biosciences	Mouse	745079	4B4-1	1:50
R-PE-labeled Pro5 MHC Pentamer A*02:01 KLPQLCTEL	Flow cytometry	PROIMMUNE	Mouse	TP/7704-03	F658-2A-G	1:50
R-PE-labeled Pro5 MHC Pentamer A*02:01 NEGATIVE	Flow cytometry	PROIMMUNE	Mouse	OP/2799-04	FN01-2A-G	1:50
Anti-human PD-L1	Immunohistochemistry	Abcam	Rabbit	ab213524	EPR19759	1:250
Anti-human TOX	Immunohistochemistry	Abcam	Rabbit	ab155768	EPR32578	1:500
Anti-human CD56	Immunohistochemistry	Cell Signaling Technology	Rabbit	99746T	E7X9 M	1:500
Anti-human Foxp3	Immunohistochemistry	Santa Cruz Biotechnology	Rabbit	SC-53876	2A11G9	1:1000
Anti-human CD3	Immunohistochemistry	Abcam	Rabbit	ab16669	SP7	1:1000
Anti-human CD4	Immunohistochemistry	Abcam	Rabbit	ab183685	EPR19514	1:1000
Anti-human CD8	Immunohistochemistry	Abcam	Rabbit	ab217344	EPR21769	1:500
Anti-human CD4	Immunofluorescence	Cell Signaling Technology	Rabbit	48274S	EP204	1:1000
Anti-human CD8	Immunofluorescence	Cell Signaling Technology	Rabbit	85336S	D8A8Y	1:1000
Anti-human CD20	Immunofluorescence	Abcam	Rabbit	ab78237	EP459Y	1:1000

Supplemental Table 3. Abbreviations

Abbreviation	Definition
ACT	Adoptive cell therapy
CCRT	Concurrent chemoradiotherapy
CT	Chemotherapy
TIL	Tumor-infiltrating lymphocytes
PBMC	Peripheral blood mononuclear cell
CC	Cervical cancer
ORR	Objective response rate
HPV	Human papilloma virus
FIGO	International Federation of Gynecology and Obstetrics
EBRT	External-beam radiation therapy
RECIST	Response Evaluation Criteria in Solid Tumors
REP	Rapid expansion protocol
AEs	Adverse events
SCC	Squamous cell carcinoma
AC	Adenocarcinoma
ELISPOT	Enzyme-Linked Immunospot
GMP	Good manufacturing practices
DCs	Dendritic cells
MDSC	Myeloid-derived suppressor cells
TME	Tumor microenvironment
CR	Complete response
PR	Partial response
PD	Progressive disease
SD	Stable disease
MRI	Magnetic resonance imaging
IHC	Immunohistochemistry
IF	Immunofluorescence
UMAP	Uniform Manifold Approximation and Projection
GSEA	Gene set enrichment analysis
MANA	Mutation-associated neoantigens
TLSs	Tertiary lymphoid structures
SFU	Spot-forming units
LDH	Lactate dehydrogenase
MFI	Mean fluorescent intensity
RPMI	Roswell Park Memorial Institute
FACS	Fluorescence Activating Cell Sorter
PBS	Phosphate buffered saline
EDTA	Ethylene diamine tetraacetic acid
HPF	High-power fields
SLOs	Secondary lymphoid structures
PFS	Progression free survival
OS	Overall survival
IL-2	Interleukin-2
DEG	Differentially expressed gene
RFS	Relapse-free survival
CTAE	Common Terminology Criteria for Adverse Events
scRNA-seq	Single cell RNA sequencing
GEO	Gene Expression Omnibus
Treg	Regulatory T cells
Tex	Exhausted T cells
Tsm	T stem cells
PCs	principal components
PHA	phytohemagglutinin
SFC	Spot forming cell

Trial protocol

A Phase I Trial of Study of Adoptively transferred tumor-infiltrating lymphocyte immunotherapy following concurrent chemoradiotherapy in patients with advanced cervical cancer

FINAL PROTOCOL
Version: August 28, 2019

Principal Investigators:

Dr. He Huang	Professor Jiang Li
Department of Gynecology Oncology	Department of Biotherapy
Sun Yat-Sen University Cancer Center	Sun Yat-Sen University Cancer Center
651 Dongfeng East Road, Guangzhou, 510060. China	651 Dongfeng East Road, Guangzhou, 510060. China
Tel: 8620 8734 3105	Tel: 8620 8734 2322
Email: huangh@sysucc.org.cn	Email: lijiang@sysucc.org.cn

ClinicalTrials.gov Identifier: NCT04443296

Administration

This clinical trial protocol is intended to provide guidance and information for the conduct of the "A Phase I Trial of Study of Adoptively transferred tumor-infiltrating lymphocyte immunotherapy following concurrent chemoradiotherapy in patients with advanced cervical cancer" in participating centers. It is not for use as a guide for the management of other patients outside of the trial.

Content

1. Schema.. 3

2. Background 4

3. Objectives.. 6

3.1 Primary objectives... 6

3.2 Secondary objectives... 6

4. Subject Enrollment 6

4.1 Eligibility Criteria 6

4.2 Exclusion Criteria... 6

4.3 Criteria for Removal from Protocol Treatment 7

5. Treatment Plan 7

5.1. TIL culture techniques 7

5.2. Radiotherapy... 9

5.3. Concurrent Chemotherapy 10

6. Observation and Assessment 12

6.1. Before Treatment 12

6.2. During Treatment 12

6.3. Post-treatment Follow-up.. 13

7. Statistical Analysis.. 13

7.1. Primary Outcome Measure 13

7.2. Secondary Outcome Measures: 14

7.3. Study design, sample size and analytical approach 15

7.5. Data Management 16

7.6 Case Report Form (CRF).. 16

8. Trial Discontinuation 16

9. Ethical Considerations... 16

10. References... 16

1. Schema

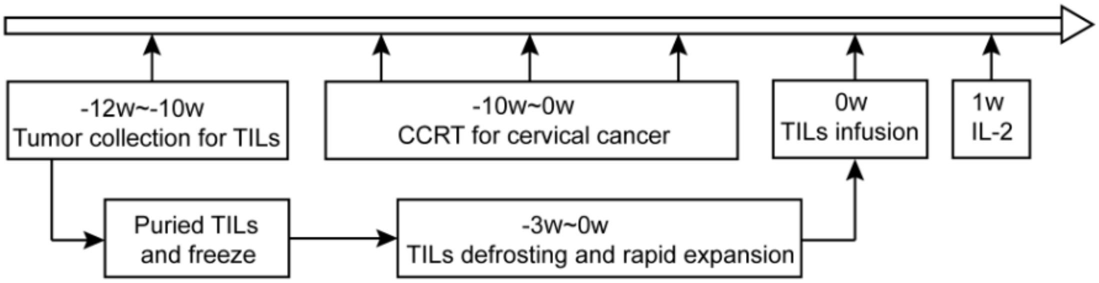

CCRT: concurrent chemoradiotherapy, TIL: tumor-infiltrating lymphocyte

2. Background

Cervical cancer (CC) is the fourth most common cancer incidence and mortality in women worldwide with about 570,000 new cases and 311,000 deaths annually[1]. Radiotherapy is the principal treatment option for patients with locally advanced or recurrent cervical cancer (tumor size large than 4cm, or FIGO stage IIB to IVA patients). To improve the outcome of treatment, the administration of chemotherapy combined with radiation treatment has significantly improved the prognosis of patients with locally advanced cervical cancer[2-5]. CCRT (concurrent chemotherapy and radiotherapy) with cisplatin has become the standard treatment for locally advanced cervical cancer. However, the improvement in long-term outcomes seems to be more pronounced for patients with stage-IB-IIB cancers compared to those with stage III and IVA cancers[6]. Once relapse, there is no effective salvage treatment after CCRT. The prognosis for stage IVA cervical cancer patients is still poor, ranging from 32-45% for 3-year OS[7, 8]. To search novel therapeutic methods combined with CCRT in primary treatment which would improve the prognosis was essential.

Adoptive cell therapy (ACT) using autologous tumor-infiltrating lymphocytes (auto-TIL) has been under development in melanoma since the 1980s, which can induce complete tumor responses in some patients[9-12]. Recently, the TIL-based ACT has been used in HPV-positive oropharyngeal, anal and cervical cancer patients and shown some clinical activity[13], which worth further investigation. And more evidence identified that TIL-based ACT treatment is benefit to some metastatic cancers, including some patients with checkpoint inhibition immunotherapy resistance[9, 14-16]. In clinical trials, TIL-based ACT treatment have been successful set up in metastatic cancers such as HPV-positive CC, lung cancer and melanomas, and tumor regression could be observed in some cancers, totally the ORR is from 28% to 50%[9-11, 15, 16, 17].

The role of RT and CT in immune regulation is still controversy. Some studies reported that RT can enhance TAAs presentation by DCs to immune cells, and enhanced the recruitment of anti-tumor T lymphocytes such as DCs, and CD8+ T cells in tumor site by up regulating adhesion molecules [18]. On the other hand, RT can directly inactivate immune cells, and leads to the recruitment of MDSCs and Treg cells in TME, promoting immune tolerance towards tumor cells[19, 20]. In addition, our previous Phase I study of CCRT combined TIL in NPC have showed the CCRT could induce lymphodepletion. In this study, CCRT was also set as a lymphodepletion treatment prior to TIL infusion in consider of the rationality of the overall treatment scheme and the toxicity of lymphodepletion regimen. A significant decrease of lymphocyte count and relieve of immunosuppression (decrease of Treg cells) was observed after CCRT. Therefore, it is a reasonable modality with CCRT followed by immnotherapy such as ACT infusion.

We have been established the primary treatment pattern that TIL-based ACT combined with CCRT in EBV-positive nasopharyngeal carcinoma patients with advanced disease stage, the objective clinical response and EBV-specific reactivity of T cells were observed in some patients[21]. And we also have set up an *ex vivo* 'Young' TIL expansion method under standard 'GMP' condition from transvaginally biopsy of tumor tissues.

In this clinical trial, we sought to further investigate the safety, feasibility and clinical response of this TIL-based ACT following CCRT in CC patients with advanced disease stage (FIGO stage IIIA-IVA). Correlations between immune parameters (including the levels of immune suppressive factors and infiltrated immune subsets in tumor tissues, and lymphocyte subset composition and HPV-E6/7 specific T cell frequency in infused TILs and peripheral blood, and the transcriptome characteristics of infused TILs, as well as the serum inflammatory cytokine profile) and clinical response were evaluated, to screen the potential predictors for the clinical benefit of TIL-based ACT.

3. Objectives

3.1 Primary objectives

Primary objective of the study was to evaluate the safety of auto-TIL following CCRT in treating patients with FIGO stage IIIA to IVA cervical carcinoma. Toxicity profile and severe toxicity were evaluated according to the Common Terminology Criteria for Adverse Events (CTCAE), version 5.0.

3.2 Secondary objectives

Secondary objectives included feasibility, primarily tumor response and its association with immunologic parameters, progression free survival (PFS) and overall survival (OS). Tumor response included response rate (ORR, including complete response and partial response), disease control duration.

4. Subject Enrollment

4.1 Eligibility Criteria

- Patients with histologically proven squamous cell carcinoma, adenocarcinoma or adenosquamous carcinoma of the cervical uteri
- Patients with stage IIIA-IVA cervical cancer according to International Federation of Gynecology and Obstetrics (FIGO) stage system 2018
- Patients who were ≥ 18 and ≤ 70 years old
- Patients with an ECOG performance status of 0, 1, or 2
- Adequate hematological, renal, and hepatic functions defined as:
- granulocytes ≥ 1.5 x 109/L, platelets ≥ 100 x 109/L, total bilirubin, ALT and AST ≤ 1.5 x upper normal limit, creatinine ≤ upper normal limit
- Patients provided written, voluntary informed consent
- Patients who were accessible to follow up and management in the treatment center

4.2 Exclusion Criteria

- Patients with past or current history of malignancy other than the entry diagnosis except for a "cured" malignancy more than five years prior to enrollment
- Patients who received previous chemotherapy or radiotherapy
- Patients with active angina or documented myocardial infarction within the 6 months preceding registration and patients with a history of significant ventricular arrhythmia requiring medication or congestive heart failure, as well as a history of 2nd or 3rd degree heart blocks
- Patients with an active infection or other serious underlying medical conditions that would impair the ability of the patient to receive the planned treatment, including prior allergic reactions to drugs containing platinum
- Patients with dementia or altered mental status that would prohibit the understanding and providing of informed consent
- Patients with inadequate caloric and/or fluid intake

4.3 Criteria for Removal from Protocol Treatment

- Serious adverse events or intolerable toxic side effects of treatment (approved by PI)
- Withdrawal of informed consent

5. Treatment Plan

Patients were treated with external-beam radiotherapy (EBRT) to a dose of 45 Gy for the primary tumor and regional lymphatics at risk. The primary cervical tumor was then boosted using brachytherapy, with an additional 30 to 40 Gy for a total point A dose of ⩾ 85 Gy. During EBRT, cisplatin was given weekly at 30 to 40 mg/m2 for a maximum of 6 doses. Ex vivo expanded auto-TILs (200 ml at concentration range in 10^7~10^8/ml) were infused 3 days after the completion of CCRT and brachytherapy. After cell infusion, interleukin-2 (IL-2) was administered as an IM bolus at 400,000 IU/dose every 24 hours to 7 doses

5.1. TIL culture techniques

5.1.1. Reagents and supplies

The preparation of TILs requires the following materials:

- Recombinant human interleukin-2 for injection (clinical use of standards; Shanghai Huaxin Biological High-tech Co., Ltd., Shanghai, China)
- OKT3, anti-CD28 (R & D Company, Minneapolis, MN, USA)
- Serum-free medium: VIVO, (Cat# 04-418Q; LONZA, Basel, Switzerland)
- Human lymphocyte separation medium (Cat# LTS1077; clinical use standard; Tianjin Hao Yang Biological Products Technology Co., Ltd., Tianjin, China)
- 0·9% saline for injection (clinical use standard)
- Gentamicin for injection
- Heparin sodium for injection
- The cell culture plates, flasks, pipettes, and all consumables to be used in the culture process are disposable. The abovementioned reagents will be purchased by the hospital pharmacy.
- Human peripheral blood lymphocytes will be obtained from healthy blood donors and provided by the central blood bank.

5.1.2. Preparation of CC TILs

- Fresh tumor tissue from the uterus cervix biopsy specimens obtained before treatment will be collected and placed in 15- or 50-mL sterile test tubes containing 10 mL serum-free RPMI1640 with antibiotic.
- After cutting the tumor tissue under normal working conditions at a clean bench, the tumor tissue will be digested with serum-free RPMI1640 and type IV collagenase (0.1 mg/ml) at 37°C for 2–3 hours (depending on the size of the tumor).
- After digestion, centrifugation, and discarding of the supernatant, the cell sediment will be washed 1–2 times with serum-free RPMI1640.
- The cells will be suspended in VIVO supplemented with 1000 IU/mL recombinant human interleukin-2 and cultured in 12-well cell culture plates. The medium and

wells will be changed whenever necessary. The cells will be cultivated for 2–3 weeks (14–22 days, as different tumors and patients may have differences in time to culture growth), until the tumor cells are all dead and the TIL count is >10^7. Subsequently, the "Young TILs" will be harvested for large-scale amplification or cell cryopreservation.

- Amplification of young TILs: 0·5–1 × 10^6 TILs will be co-cultured with irradiated (40 Gy, X-ray) allogeneic PBMCs (50–200-fold) in a 175-mm^2 flask with VIVO containing IL-2 3000 IU/mL, OKT-3 30 ng/mL, and anti-CD28 antibody 30 ng/mL. The liquid will be changed on the 5th and 8th day and the culture of cells will be expanded when necessary every day after the 8th day. Once the number of cells increases, the cells can be transferred to culture bags. After 15 days of rapid amplification, the number of cells could reach 10^9–10^{10} (the number of cells will vary among tumors and patients). The total cell culture time will be approximately 35 days (from tumor biopsy to cell transfusion).
- The TIL samples will be inspected 1 day before expansion and 2 days before transfusion, and 1 mL will be extracted per bottle. After the details are described in an inspection sheet, the detection of bacterial and fungal organisms will be performed in the Bacteriology Room of the Laboratory Department.
- On day 15, we will assess the laboratory results of the bacterial and fungal test. After observing the state of the cell growth, 5 mL of the culture will be randomly and uniformly sampled, and a phenol blue staining method would be used to count the total number of living and dead cells. After ensuring that the cell suspension is negative for endotoxin, the cells will be subsequently collected in a sterile 250-mL centrifuge cup after centrifugation at 2500 rpm for 8 min.
- The supernatant will be discarded, and the cells will subsequently be washed twice with 0·9% sterile saline and centrifuged at 2000 rpm for 8 min.
- The supernatant will be discarded, and the cells will be collected in approximately 150 mL 0·9% sterile saline. Subsequently, 5 mL of 20% human serum albumin will be extracted with a 5-mL syringe into the cell suspension, and 1 mL of cell suspension will be sampled with a graduated pipette and sealed in a glass ampoule. The sample will be stored at 4°C for 3 months.
- Finally, the finished cell suspension will be sealed in a bottle labeled with the patient's ward, name, hospital number, sex, age, cell count, endotoxin, test results of sterility, and other items to ensure correct labeling; the detailed experimental records and signatures will be completed; and the suspension will be reinfused back into the patients.

5.1.3. TIL infusion and Toxicity monitoring

Patients will receive auto-TILs infusion (200 ml at concentration range in 10^7~10^8/ml) 3 days after the completion of radiotherapy. A low dosage of IL-2 (at 400,000 IU) will be injected daily intramuscularly for 1 week after the transfusion. Signs and symptoms of acute toxicity reaction, including an allergic reaction, will be observed for 6 hours after reinfusion. Treatment-related toxicity effects will be evaluated till 4 weeks after the treatment.

5.1.4 TIL quality control (SOP)

The performance of the equipment and experimental preparations are consistent with the safety standards for clinical use. The final formulation of TILs must meet the following requirements, and assessments will be occasionally performed, typically twice weekly.

- The cell survival rate should be more than 95%, and the cell phenotype must meet the

following requirement: >90% CD3+ cells;

- Live cell count >5×10^8;
- Absence of exogenous factors: bacteria, fungi, mycoplasma, viruses, and endotoxins; sterility tests would be undertaken on the 1st and 5th day of preparation and 30 hours before the collection, and would be performed in the Laboratory Department.;
- Absence of other added ingredients (i.e., serum proteins, antibodies, serum, antibiotics, and solid particles).

5.2. Radiotherapy

5.2.1. Radiation treatment planning

All patients enrolled would be treated with intensity-modulated radiotherapy (IMRT). Patients were treated with external-beam radiotherapy (EBRT) to a dose of 45 Gy for the primary tumor and regional lymphatics at risk. The primary cervical tumor was then boosted using brachytherapy, with an additional 30 to 40 Gy for a total point A dose of ⩾ 85 Gy.

EBRT:

Fractionation schedule

Pelvic radiation will be applied in fractions of 1.8 Gy or 2.0 Gy daily, five times a week with a total dose of 45-50 Gy. All patients will be radiated by external beam radiation.

Position of the patient

The patient will be positioned in supine position.

Definitions of target volumes and critical structures

The radiation field is as follow: 2cm lateral to the bony margin of the pelvis, up to L5 and S1 vertebral body, 2cm below the obturator foramen. And intracavitary boost 30-35Gy/4 weeks was conducted if with positive vaginal margins only. In case of tumor involvement of the common iliac lymph nodes, the radiation field will exptend up to the 3/4 lumbar vertebral body.

Simulation procedure

Prior to the start of the irradiation a planning CT scan will be made with a slice thickness of 5 mm, with the patient in treatment position. The isocenter will be determined at the planning-CT.

External beam equipment

Radiation therapy will be delivered with megavoltage equipment with photon energies of equal to or greater than 6 MV.

Dose specification

The prescription dose will be specified at the ICRU 50/62 reference point, which will be the isocenter for most patients. The daily prescription dose will be 1.8 Gy or 2Gy at the ICRU reference point and the 95% isodose must encompass the entire planning target volume (PTV). The maximum to the PTV must not exceed the prescription dose by >7% (ICRU 50/62 guidelines). Tissue density inhomogeneity correction will be used.

Treatment verification

Portal images will be obtained during the first fraction of all fields. On indication portal images will be repeated.

Patient monitoring

Pelvic radiotherapy has both short and long-term side effects. The immediate side effects include nausea, fatigue, cystitis, acute small bowel reactions and proctitis. Later side effects include telangiectasia of the bladder and bowel, chronic proctitis and tiredness in association with frequency, urgency. Damage to the rectal mucosa can cause long-term rectal blood loss.

5.2.2. Evaluation and grading according to the Common Terminology Criteria for Adverse Events (CTCAE v5.0) toxicity criteria:

- Radiotherapy adjustment in the presence of hematological toxicity

Radiation treatment will be performed only if the absolute neutrophil count (ANC)is ⩾0·5 × 109/L and PLT count is ⩾50 × 10^9/L

- Radiotherapy adjustment in the presence of non-hematological toxicity

Side effects of radiotherapy may include short and long-term side effects. The immediate side effects include nausea, fatigue, cystitis, acute small bowel reactions and proctitis. Later side effects include telangiectasia of the bladder and bowel, chronic proctitis and tiredness in association with frequency, urgency. Damage to the rectal mucosa can cause long-term rectal blood loss. Interruption of radiotherapy may occur if there is a persistent and irreducible severe side effect of radiotherapy (assessed and reported by the clinician).

5.3. Concurrent Chemotherapy

5.3.1. Concurrent chemoradiotherapy

Cisplatin (DDP) of 30~40 mg/m^2 will be given intravenously weekly during EBRT (totally 5 cycles and no more than 6 cycles).

Premedication

The proper prophylaxis for cisplatin-based chemotherapy regimens is a 5HT3 receptor antagonist (ondansetron, granisetron, and dolasetron) in combination with

dexamethasone, usually 10mg administered 30 minutes prior to starting chemotherapy.

Administration of cisplatin

Cisplatin diluted in NaCl 0.9% 500ml will be infused over one hour. Additional intravenous hydration after the cisplatin with a total of at least 1 liter of IV normal saline is recommended.

Patient monitoring

Cisplatin may potentiate renal toxicity and should be avoided whenever possible. Severe renal toxicity can be largely avoided by induction of a diuresis before, during and after treatment.

Adverse effects: Leukopenia, thrombocytopenia, anemia, nausea, vomiting, nephrotoxicity, ototoxicity, peripheral neuropathy, electrolyte imbalance, hypocalcemia, hypomagnesemia, aminoglycoside ototoxicity, ocular toxicity, and allergic reactions.

Infrequent: Cardiac abnormalities, anorexia, elevated SGOT, rash, alopecia, and acute myeloid leukemia.

Drug Reactions

Adverse drug reaction occurred while being infused can be infusion or allergic reaction. Drug reactions will be classified as mild, moderate or severe.

Even the patient had mild reaction while being infused with cisplatin will develop serious reaction, the infusion of cisplatin should be stopped immediately. The reuse of cisplatin should be fully discussed with patient and with emergency equipment or considered consultation with allergist.

5.3.2. Discontinue with cisplatin

- **Discontinue for hematological toxicity**

If the WBC are < 1.0 × 10^9/L and/or platelets < 50 × 10^9/L: delay chemotherapy by 1 week until recovery above these values. In the event of grade 4 adverse events or febrile neutropenia (granulocyte < 0.5 × 10^9/L/L and fever >38.5 ^{0}C) both chemotherapy and radiotherapy will be withheld until the recovered.

- **Discontinue for non-hematological toxicity**

- Renal toxicity

Reaction	Management of reaction
Creatinin <=1.5 x the upper limit of normal at the day of retreatment	Continue therapy
Creatinin is > 1.5 x the upper limit of normal.	> 1.5 x the upper normal limit → Stop chemotherapy.

- Neurologic toxicity:
  In patients with grade 3 neurotoxicity, DDP should be discontinued.

- Gastrointestinal toxicity:
  In the event of grade 3 adverse events including myelosuppression or gastrointestinal events chemotherapy will be withheld until recovered.

6. Observation and Assessment

6.1. Before Treatment

- Medical history
- The present medications and treatment
- Physical examination, including height, weight, and vital signs; gynecological examination
- ECOG score
- Chest X-rays or contrast-enhanced CT
- Abdominal and pelvic cavity contrast-enhanced MRI/CT
- Positron emission tomography–computed tomography (PET/CT) if economically feasible
- ECG
- Blood biochemistry (e.g., liver function, kidney function, electrolytes)
- Blood routine (white blood cells, neutrophils, PLT, hemoglobin)
- Urine routine
- HPV examination (HPV DNA detection from tans-vaginal collection)
- Blood sample
- Eligibility for identification and informed consent
- Collection of fresh tumor tissue and blood specimens from patients undergoing trans-vaginal biopsy of lesion in uterus cervix before treatment

6.2. During Treatment

6.2.1. Evaluation from the beginning to the end of treatment

- Toxicity: adverse Evaluation of adverse effect based on the standards of the National Cancer Institute (NCI)-CTCAE 4.0, including hematological toxicity, gastrointestinal reactions, liver and kidney dysfunction.
- TIL immunotherapy: Observing the patient's vital signs before and after infusion, and simultaneously detecting the immunological index HPV, including antigen-specific CTLs, lymphocyte subsets, and immune suppression cell subsets.
- The use of other drugs.
- General medical situation.
- Laboratory examination: Blood routine and biochemical tests before and 1 month after CCRT, Blood routine weekly during CCRT.

- Evaluation of the tumor: Weekly Physical and gynecological examination during CCRT.
- Collection of fresh tumor tissue and blood specimens from patients undergoing trans-vaginal biopsy of lesion in uterus cervix before TIL infusion

6.2.2. Evaluation During Radiotherapy

From the beginning to the end of concurrent chemo-radiotherapy, the adverse effects related to radiotherapy will be evaluated according to the CTCAE 5.0

6.3. Post-treatment Follow-up

6.3.1 Post-treatment evaluation

- ECOG score
- Blood routine
- Biochemical routine
- Blood samples to be obtained at the end of CCRT and at 1, 3, and 6 months after treatment
- Recording of adverse effects
- Physical and gynecological examination, and performance of a therapeutic evaluation
- Contrast-enhanced MRI/CT/PET-CT of pelvic and abdominal cavity every 3 month after treatment until recurrence or progression
- Chest lateral radiograph or chest CT

6.3.2 Follow-up and recording of events

After treatment, patients were followed up every 3 months during the first 2 years and every 6 months thereafter. Investigations with MRI/CT/PET-CT should be arranged every 3 months after the first year after treatment, then every 6 month in the following second year. Treatment responses were evaluated according to the RECIST criteria (version 1.1). If residual disease was found, the modalities of salvage treatments (radiotherapy, surgery, and/or chemotherapy) will be decided by the clinician when necessary. Failure should preferably be confirmed by a biopsy or fine-needle aspiration whenever possible. The clinical diagnosis was established when classic changes occurred on imaging methods, including PET-CT, MRI, CT, chest X-ray, bone scanning, with a concordant clinical development.

7. Statistical Analysis

7.1. Primary Outcome Measure

- **Toxicities**

Toxicities will be evaluated by recording clinical adverse events (AEs) using the National Cancer Institute Common Toxicity Criteria (NCI-CTCAE version 5.0). All patients will be evaluable for toxicity from the begin of radiotherapy to 30 days after the complete of all assigned treatments. Severe toxicity was defined as any of the following AE/SAE as assessed in the reporting period by the investigator: Any grade > 3 (non-autoimmune)

toxicity suspected to be related to the TIL infusion (not related to cervical cancer or other pre-existing condition in CCRT), any grade 3 autoimmune event that did not resolve with intervention (steroids), to a grade 1 or less within 21 days.

7.2. Secondary Outcome Measures:

- **Feasibility**

Feasibility was defined as the rate of successful TIL generation from tumor biopsy specimens.

- **Tumor Response**

Disease response is evaluated based upon timepoints after the adoptive CCRT and auto-TIL infusion, and the tumor size evaluated using the RECIST 1.1 criteria including a complete response (CR), partial response (PR), stable disease (SD) or progressive disease (PD). Complete response is defined as the complete disappearance of the target and non-target lesion(s) identified at baseline after radiological evaluation.

Evaluations will be made for patients by MRI/CT/PET-CT scan approximately every 3 months after the treatment, and by clinical evaluation during this time. The objective response (CR+PR) rate (ORR) will be summarized using both a point estimate and its exact 95% confidence interval based on the binomial distribution. Disease control (CR+PR+SD) rate (DCR) was also calculated. Disease control time (DCT) was defined as duration from the time of objective response to tumor progression or last follow up.

- **Progression free survival (PFS) and Overall survival (OS)**

PFS and OS were defined as the time from treatment initiation until progression or death from any cause, respectively, or the date of data cutoff.

- **Serum cytokine profile analysis and Immunochemistry and immunofluorescence**

Serum cytokine profile analysis

Serum cytokine and chemokine levels in serum were measured using a cytokine Milliplex assay kit (Millipore Sigma) and MAGPIX Multiplexing System (Millipore Sigma) following the manufacturer's protocol.

Immunochemistry and immunofluorescence

Formalin-fixed paraffin-embedded tissue sections were continuously sectioned at a thickness of 4 μm, and an immunohistochemistry kit (Zhongshanjinqiao) was used according to the manufacturer's instructions. In brief, tissue sections were deparaffinized and rehydrated by immersion in EDTA (pH 8.0) or 1x citrate (pH 6.0). A pressure cooker (95 °C 22 min) was applied for antigen retrieval. Goat serum was applied to block nonspecific binding sites at room temperature for 30 min. Primary antibodies, including anti-human PD-L1, anti-TOX, anti-Foxp3, and anti-CD56 antibodies, were incubated at 4° C overnight. The secondary antibody (Zhongshanjinqiao) was incubated for 30 min at

room temperature. 3,3′-Diaminobenzidine tetrahydrochloride (DAB) was used for visualization. Finally, the pathological sections were counterstained with hematoxylin, dehydrated and sealed with neutral glue for optical microscopy.

For immunofluorescent staining of TILs, an Opal plorarisTM 7 color manual IHC kit (Akoya Biosciences) was used following the manufacturer's protocol with primary antibodies, including anti-human CD4, CD8 and CD20 antibodies. DAPI was used for nuclear staining and section mounting. Images were acquired using a PerkinElmer Vectra V.3.0 system, and Vectra software (Akoya Biosciences, Marlborough, Massachusetts) and HALO software (Indica Labs, Albuquerque) were used to analyze all images. Lymphocyte density was quantified as % of cells expressing a given marker of at least 5 high-power fields (HPFs). Mature TLSs correspond to lymphoid follicles, including a dense cellular aggregate resembling germinal centers found in secondary lymphoid structures (SLOs). The calculation of the immune combined score was as follows: 0 = low expression of PD-L1, TOX, or Foxp3 or high expression of CD4, CD8, CD20, CD56, or TLS; 1 = high expression of PD-L1, TOX, or Foxp3 or low expression of CD4, CD8, CD20, CD56, or TLS. The score was calculated by adding the expression of individual markers. The maximum score was 8.

7.3. Study design, sample size and analytical approach

This trial is a single-center, phase I study that was designed to investigate the safety, feasibility and preliminary tumor response, translational investigations for adjuvant immunotherapy using infusion of autogenous (auto)-TILs following concurrent chemoradiotherapy (CCRT) in patients with International Federation of Gynecology and Obstetrics (FIGO) stage IIIA to IVA cervical carcinoma. We aim to evaluated 12 patients for the toxicity in this study. Every 3 patients were treated as a cohort and evaluated for the toxicity. If no dose severe toxicity or 1 severe toxicity was observed in the first 3 patients, then 3 more patients were enrolled into the next cohort till 12 patients were included. If 2 or more patients within a cohort experienced severe toxicities, then that the study will be stopped. Severe toxicity was defined as any of the following AE/SAE as assessed in the reporting period by the investigator: Any grade > 3 (non-autoimmune) toxicity suspected to be related to the TIL infusion (not related to cervical cancer or other pre-existing condition in CCRT), any grade 3 autoimmune event that did not resolve with intervention (steroids), to a grade 1 or less within 21 days. Toxicity will be assessed within 30 days of the adoptive TIL transfer. A maximum of 30 patients will be enrolled to obtain 12 evaluable patients for the safety assessment. Being evaluable is defined as patients that receive CCRT and auto-TIL infusion and were assessable for toxicity in 30 days after the treatments.

The data analysis will mainly be descriptive, with inferential statistics provided for the primary and secondary endpoints. Summary statistics for adverse events, including the proportions of each preferred adverse event type will be tabulated and assembled into Tables. Adverse events will be categorized by Grade. The categorical variables are reported as the number of cases and frequency (%). All correlative study results will be treated as exploratory in nature due to the pilot status and sample size of the trial. Patients who are lost to follow-up or drop out of the study prior to their scheduled evaluation time due to any reason will be included for feasibility determination.

Baseline characteristics such as patient age, histology, and HPV status will be summarized in a descriptive table including relevant average. This secondary endpoint will be reported descriptively. Objective response will be plotted on applicable 'Waterfall' plots using percent change from nadir = (total length – nadir in total length) / (nadir in total length) x 100%. For correlative analysis, we will explore the extent to which changes between pre- and post-treatment levels correlate with response by t-test or Mann–Whitney U test. The significance of differences in values between time points was tested with the Wilcoxon signed rank test for nonparametric data. This will be done on the percent change from the pre-treatment values for the biomarkers at $\alpha = .05$, reported only for exploratory testing.

7.5. Data Management

All patients will be included and treated in our hospital. After registration, the information about enrolled patients will be recorded. We will have stewards taking charge of database management.

7.6 Case Report Form (CRF)

The CRF will be designed before the study and is required to record detailed information on medical history, treatment, and follow-up, and it should be easy to fill in and store in the database.

8. Trial Discontinuation

For reasonable cause the investigator may terminate this study prematurely. Conditions that may warrant termination include: the discovery of an unexpected, significant, or unacceptable risk to the patients enrolled in the study or if the accrual goals are met.

9. Ethical Considerations

This clinical study protocol was approved officially by the Ethics Committee of the Cancer Center of Sun Yat-sen University. According to the GCP guidelines, any modification of the research protocol will require the consent of the Ethics Committee. This clinical study follows the principles of the Declaration of Helsinki and the relevant clinical research norms and regulations applicable in People's Republic of China. Before the subject is enrolled in the study, the investigator must provide a full description of the purpose, procedure, and possible risks of the clinical study to the subject or the subject's legal representative. Subjects must be made aware that they have the right to withdraw from the study at any time and under any circumstances. Each subject or subject's legal representative must sign an informed consent form in duplicate (one original and one copy). It is the responsibility of the study physician to obtain informed consent before each subject enters the study and to maintain the document in a research file.

10. References

1. Cohen SS, Palmieri RT, Nyante SJ, Koralek DO, Kim S, Bradshaw P, et al. Obesity and screening for breast, cervical, and colorectal cancer in women: a review. Cancer. 2008;112(9):1892-904.

2. Sorbe B, Bohr L, Karlsson L, Bermark B. Combined external and intracavitary irradiation in treatment of advanced cervical carcinomas: Predictive factors for local tumor control and early recurrences. Int J Oncol. 2010;36:371–378.
3. Morris M, Eifel PJ, Lu J, et al. Pelvic radiation with concurrent chemotherapy compared with pelvic and para-aortic radiation for high-risk cervical cancer. N Engl J Med. 1999;340:1137–1143.
4. Rose PG, Bundy BN, Watkins EB, et al. Concurrent cisplatin-based radiotherapy and chemotherapy for locally advanced cervical cancer. N Engl J Med. 1999;340:1144–1153.
5. Eifel PJ, Winter K, Morris M, et al. Pelvic irradiation with concurrent chemotherapy versus pelvic and para-aortic irradiation for high-risk cervical cancer: an update of radiation therapy oncology group trial (RTOG) 90-01. J Clin Oncol. 2004;22:872–880.
6. Vale C, Tierney JF, Stewart LA, Brady M, Dinshaw K, Jakobsen A, Parmar MKB, Thomas G, Trimble T, Alberts DS, Chen H, Cikaric S, Eifel PJ, Garipagaoglu M, Keys H, Kantardzic N, Lal P, Lanciano R, Leborgne F, Lorvidhaya V, Onishi H, Pearcey RG, Pras E, Roberts K, Rose PG, Whitney CW. Reducing uncertainties about the effects of chemoradiotherapy for cervical cancer: a systematic review and meta-analysis of individual patient data from 18 randomized trials. J Clin Oncol. 2008;26:5802–5812.
7. Rose PG, Ali S, Whitney CW, Lanciano R, Stehman FB. Outcome of stage IVA cervical cancer patients with disease limited to the pelvis in the era of chemoradiation: a gynecologic oncology group study. Gynecol Oncol. 2011;121:542–545.
8. Hata M, Koike I, Miyagi E, Numazaki R, Asai-Sato M, Kaizu H, Mukai Y, Takano S, Ito E, Sugiura M, Inoue T. Radiation therapy for stage IVA uterine cervical cancer: treatment outcomes including prognostic factors and risk of vesicovaginal and rectovaginal fistulas. Oncotarget. 2017;8:112855–112866.
9. Creelan BC, Wang C, Teer JK, Toloza EM, Yao J, Kim S, et al. Tumor-infiltrating lymphocyte treatment for anti-PD-1-resistant metastatic lung cancer: a phase 1 trial. Nature medicine. 2021;27(8):1410-8.
10. Dudley ME, Gross CA, Langhan MM, Garcia MR, Sherry RM, Yang JC, et al. CD8+ enriched "young" tumor infiltrating lymphocytes can mediate regression of metastatic melanoma. Clinical cancer research : an official journal of the American Association for Cancer Research. 2010;16(24):6122-31.
11. Kradin RL, Kurnick JT, Lazarus DS, Preffer FI, Dubinett SM, Pinto CE, et al. Tumour-infiltrating lymphocytes and interleukin-2 in treatment of advanced cancer. Lancet. 1989;1(8638):577-80.
12. Weber J, Atkins M, Hwu P, Radvanyi L, Sznol M, Yee C, et al. White paper on adoptive cell therapy for cancer with tumor-infiltrating lymphocytes: a report of the CTEP subcommittee on adoptive cell therapy. Clinical cancer research : an official journal of the American Association for Cancer Research. 2011;17(7):1664-73.
13. Stevanovic S, Helman SR, Wunderlich JR, Langhan MM, Doran SL, Kwong MLM, et al. A Phase II Study of Tumor-infiltrating Lymphocyte Therapy for Human Papillomavirus-associated Epithelial Cancers. Clinical cancer research : an official journal of the American Association for Cancer Research. 2019;25(5):1486-93.
14. Tang B, Yan X, Sheng X, Si L, Cui C, Kong Y, et al. Safety and clinical activity with an anti-PD-1 antibody JS001 in advanced melanoma or urologic cancer patients. J Hematol Oncol. 2019;12(1):7.
15. van den Berg JH, Heemskerk B, van Rooij N, Gomez-Eerland R, Michels S, van Zon M, et al. Tumor infiltrating lymphocytes (TIL) therapy in metastatic melanoma: boosting of neoantigen-specific T cell reactivity and long-term follow-up. J Immunother Cancer. 2020;8(2).
16. Besser MJ, Shapira-Frommer R, Treves AJ, Zippel D, Itzhaki O, Hershkovitz L, et al. Clinical responses in a phase II study using adoptive transfer of short-term cultured tumor infiltration lymphocytes in metastatic melanoma patients. Clinical cancer research : an official journal of the American Association for Cancer Research. 2010;16(9):2646-55.
17. Besser MJ, Treves AJ, Itzhaki O, Hardan I, Nagler A, Papa MZ, et al. Adoptive cell therapy for metastatic melanoma patients: pre-clinical development at the Sheba Medical Center. Isr Med Assoc J. 2006;8(3):164-8.

18. Hallahan, D., J. Kuchibhotla, and C. Wyble, Cell adhesion molecules mediate radiation-induced leukocyte adhesion to the vascular endothelium. Cancer Res, 1996. 56(22): p. 5150-5
19. McGinnes, K., J. Florence, and R. Penny, The effect of radiotherapy on the natural killer (NK)-cell activity of cancer patients. J Clin Immunol, 1987. 7(3): p. 210-7.
20. Bos, P.D., et al., Transient regulatory T cell ablation deters oncogene-driven breast cancer and enhances radiotherapy. J Exp Med, 2013. 210(11): p. 2435-66.
21. Li J, Chen QY, He J, Li ZL, Tang XF, Chen SP, et al. Phase I trial of adoptively transferred tumor-infiltrating lymphocyte immunotherapy following concurrent chemoradiotherapy in patients with locoregionally advanced nasopharyngeal carcinoma. Oncoimmunology. 2015;4(2):e976507.
